# Supplementary material for: Trabecular bone architecture in the stylopod epiphyses of mustelids (Mammalia, Carnivora)
Source: R Soc Open Sci. 2019 Oct 23;6(10):190938. doi: 10.1098/rsos.190938 (PMC6837213; doi:10.1098/rsos.190938)
Supplement: SM 3 [file rsos190938supp3.docx]

Supplementary Online Material for:

Trabecular bone architecture in the stylopod epiphyses of mustelids (Mammalia, Carnivora)

Amson, E.^1^ and Kilbourne, B.M.^1^

^1^Museum für Naturkunde, Leibniz-Institut für Evolutions- und Biodiversitätsforschung, Berlin, Germany

Definition of the Volumes Of Interest (VOIs) in **A-C**, the smallest (*Mustela erminae* ZMB_Mam 69395) and **D-G**, largest *(Enhydra lutris* ZMB_Mam 30740 and ZMB_Mam 82975) taxa of the dataset. See also Figure 2 of main text. **A**, side of humeral head VOI = 1.35 mm, side of humeral trochlea VOI = 0.63 mm; **B**, side of femoral head VOI =1.53 mm; **C**, side of lateral condyle of the femur VOI = 1.35 mm; **D**, side of humeral head VOI = 3.22 mm; **E**, side of humeral trochlea VOI = 2.74 mm; **F**, side of femoral head VOI = 8.73 mm; **G**, side of lateral condyle of the femur VOI = 6.63 mm).
