# Supplementary material for: Trabecular bone architecture in the stylopod epiphyses of mustelids (Mammalia, Carnivora)
Source: R Soc Open Sci. 2019 Oct 23;6(10):190938. doi: 10.1098/rsos.190938 (PMC6837213; doi:10.1098/rsos.190938)
Supplement: SM 5 [file rsos190938supp5.docx]

Supplementary Online Material for:

Trabecular bone architecture in the stylopod epiphyses of mustelids (Mammalia, Carnivora)

Amson, E.^1^ and Kilbourne, B.M.^1^

^1^Museum für Naturkunde, Leibniz-Institut für Evolutions- und Biodiversitätsforschung, Berlin, Germany

Descriptive account of the main direction of the trabeculae (MDT).

The main direction of the trabeculae (MDT) does not show clear differences among the locomotor specializations (Fig. SOM3). For all specimens the MDT of the humeral head is directed distoanteromedially (Fig. SOM3A). For the femoral head, most of the specimens show MDT close to the proximodistal axis, with a few species deviating towards a more transversal orientation (Fig. SOM3C). Both distal ROIs (humeral and femoral) show a similar MDT pattern, with specimens’ orientation found along an anteromedial-posterolateral axis (Fig. SOM3B, D). While a slight separation among the specialization types can be observed for the humeral head MDT, there is a considerable amount of overlap ruling out their clear discrimination. Any distinction is even less clear for the other investigated ROIs.

Figure SOM3 (next page). Main direction of the trabeculae (MDT) in the humeral and femoral epiphyses of mustelids. A, humeral head; B, humeral trochlea; C, femoral head; lateral condyle of the femur. =>
