# Supplementary material for: Trabecular bone architecture in the stylopod epiphyses of mustelids (Mammalia, Carnivora)
Source: R Soc Open Sci. 2019 Oct 23;6(10):190938. doi: 10.1098/rsos.190938 (PMC6837213; doi:10.1098/rsos.190938)
Supplement: SM 6 [file rsos190938supp6.pdf]

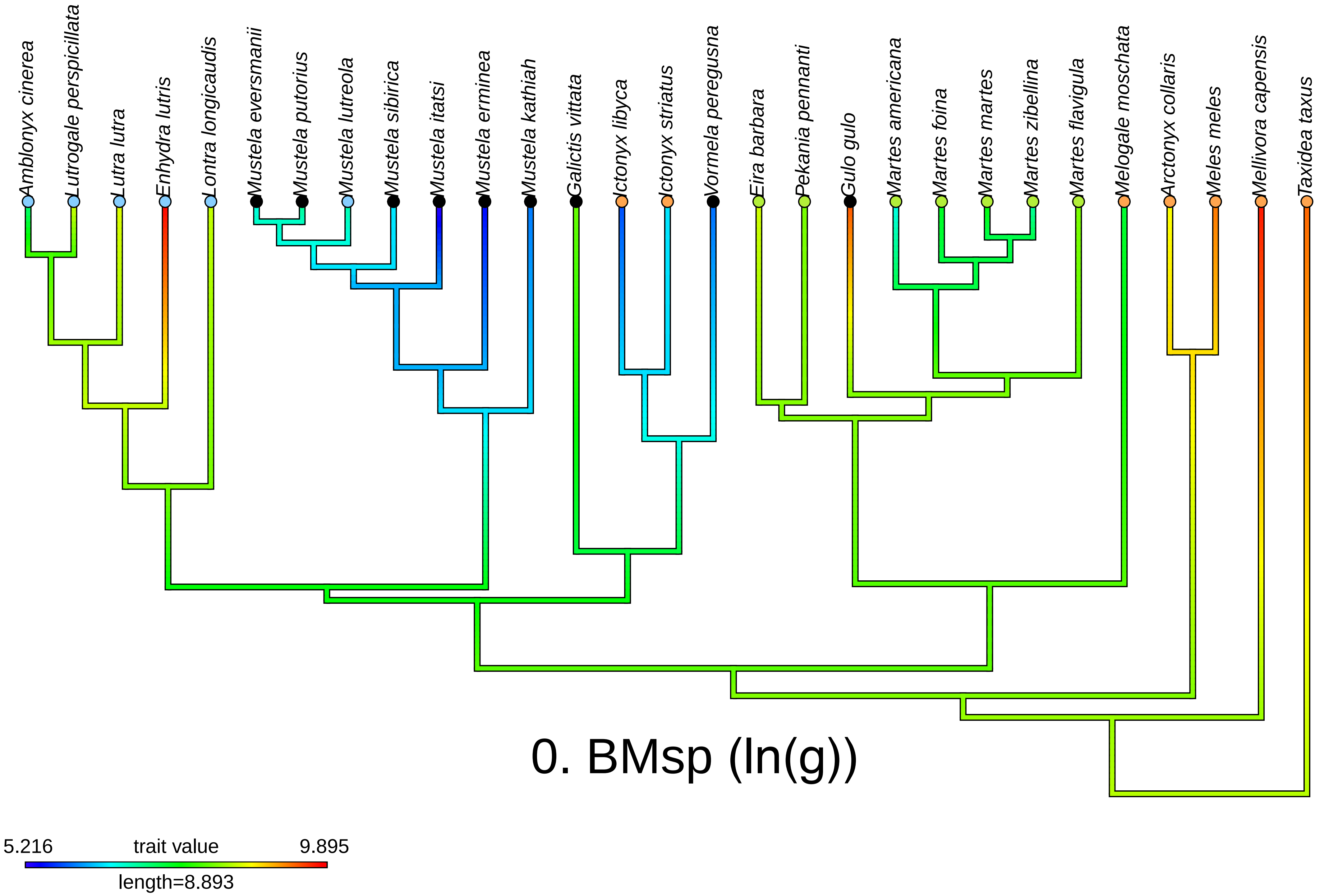
Amblonyx cinerea
Lutrogale perspicillata
Lutra lutra
Enhydra lutris
Lontra longicaudis
Mustela eversmanii
Mustela putorius
Mustela lutreola
Mustela sibirica
Mustela itatsi
Mustela erminea
Mustela kathiah
Galictis vittata
Ictonyx libyca
Ictonyx striatus
Vormela peregusna
Eira barbara
Pekania pennanti
Gulo gulo
Martes americana
Martes foina
Martes martes
Martes zibellina
Martes flavigula
Melogale moschata
Arctonyx collaris
Meles meles
Mellivora capensis
Taxidea taxus
5.216
trait value
9.895
length=8.893

0. BMsp (ln(g))

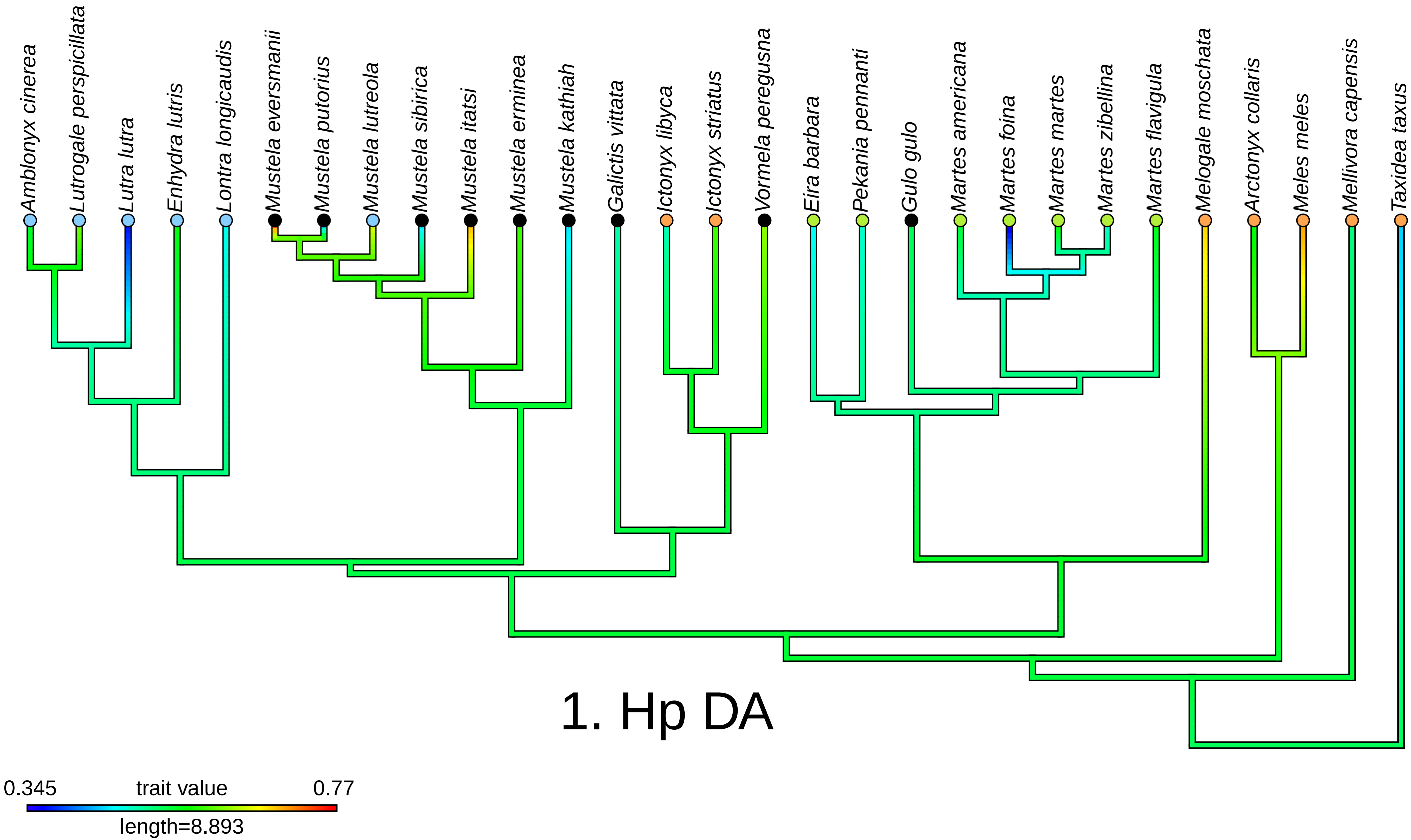
Amblonyx cinerea
Lutrogale perspicillata
Lutra lutra
Enhydra lutris
Lontra longicaudis
Mustela eversmanii
Mustela putorius
Mustela lutreola
Mustela sibirica
Mustela itatsi
Mustela erminea
Mustela kathiah
Galictis vittata
Ictonyx libyca
Ictonyx striatus
Vormela peregusna
Eira barbara
Pekania pennanti
Gulo gulo
Martes americana
Martes foina
Martes martes
Martes zibellina
Martes flavigula
Melogale moschata
Arctonyx collaris
Meles meles
Mellivora capensis
Taxidea taxus
0.345
trait value
0.77
length=8.893

1. Hp DA

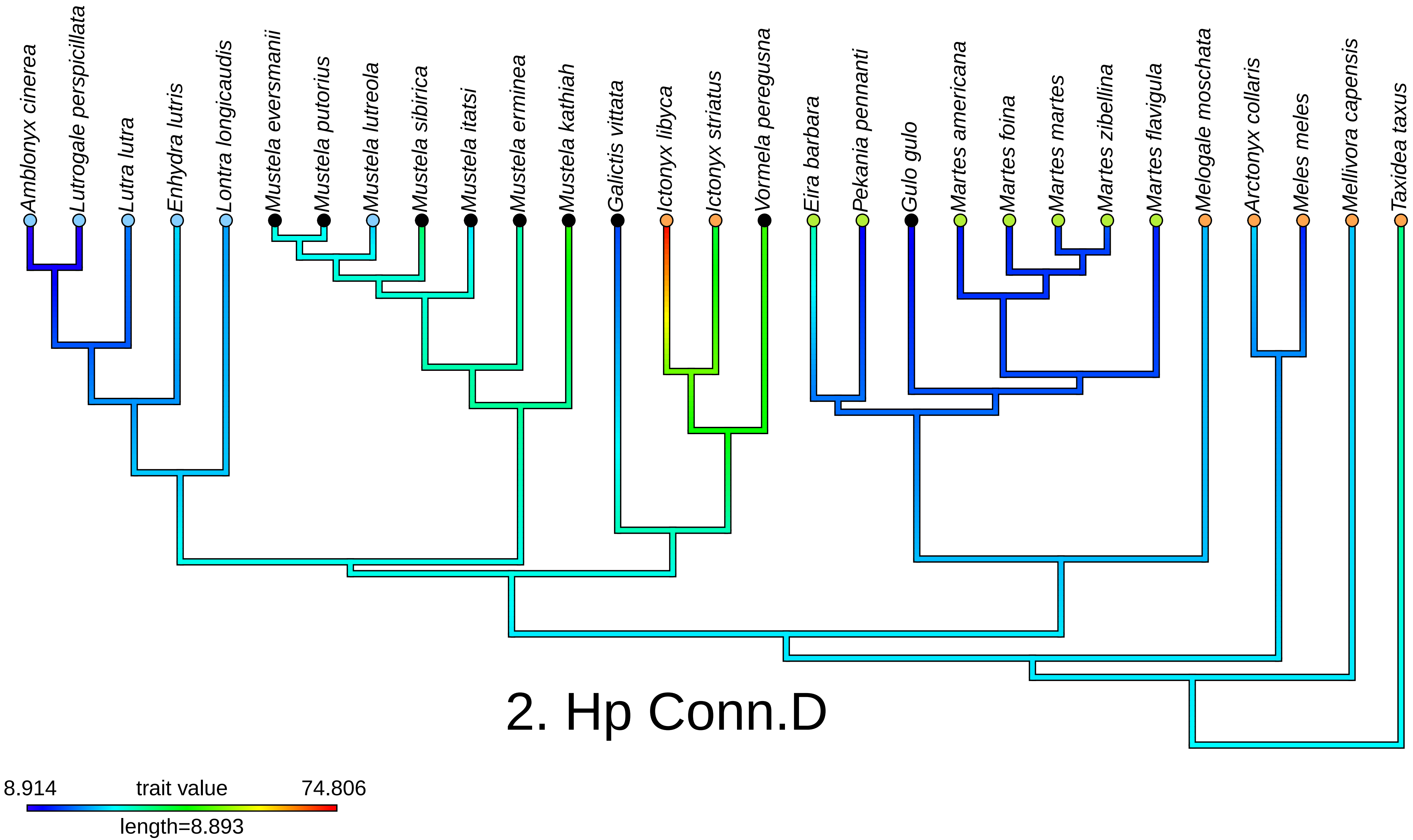
Amblonyx cinerea
Lutrogale perspicillata
Lutra lutra
Enhydra lutris
Lontra longicaudis
Mustela eversmanii
Mustela putorius
Mustela lutreola
Mustela sibirica
Mustela itatsi
Mustela erminea
Mustela kathiah
Galictis vittata
Ictonyx libyca
Ictonyx striatus
Vormela peregusna
Eira barbara
Pekania pennanti
Gulo gulo
Martes americana
Martes foina
Martes martes
Martes zibellina
Martes flavigula
Melogale moschata
Arctonyx collaris
Meles meles
Mellivora capensis
Taxidea taxus
8.914
trait value
74.806
length=8.893

2. Hp Conn.D

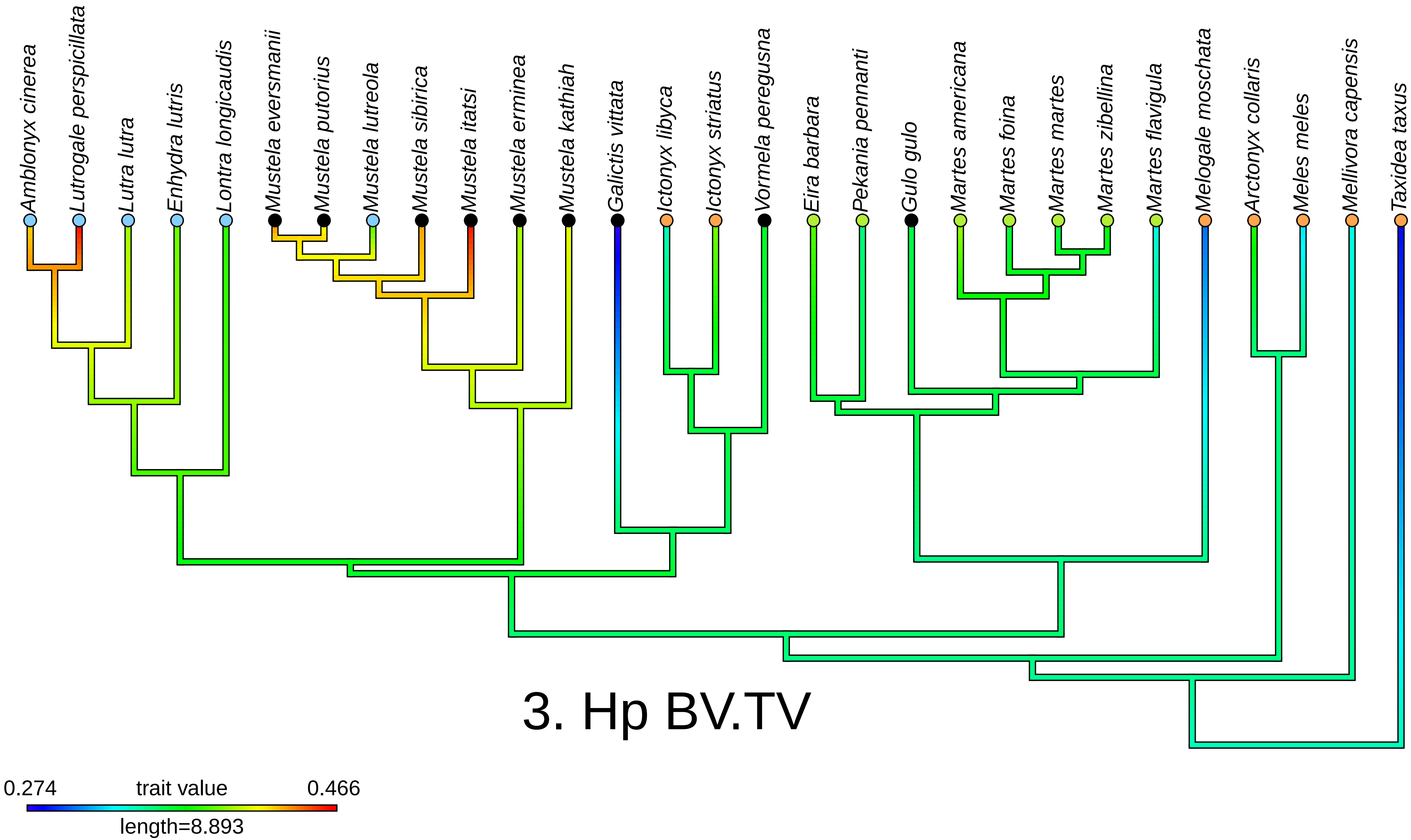
Amblonyx cinerea
Lutrogale perspicillata
Lutra lutra
Enhydra lutris
Lontra longicaudis
Mustela eversmanii
Mustela putorius
Mustela lutreola
Mustela sibirica
Mustela itatsi
Mustela erminea
Mustela kathiah
Galictis vittata
Ictonyx libyca
Ictonyx striatus
Vormela peregusna
Eira barbara
Pekania pennanti
Gulo gulo
Martes americana
Martes foina
Martes martes
Martes zibellina
Martes flavigula
Melogale moschata
Arctonyx collaris
Meles meles
Mellivora capensis
Taxidea taxus
3. Hp BV.TV
0.274
trait value
0.466
length=8.893

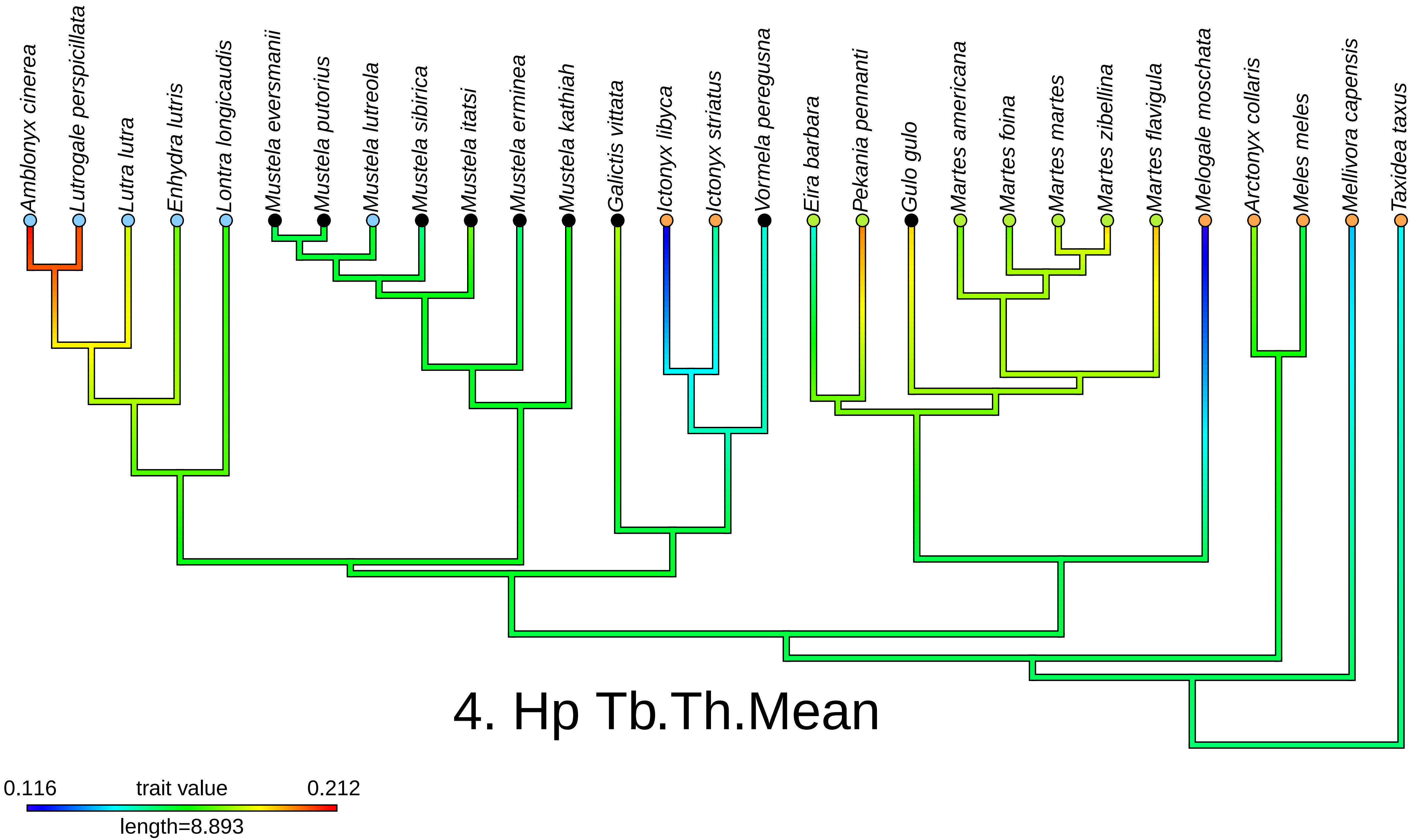

4. Hp Tb.Th.Mean

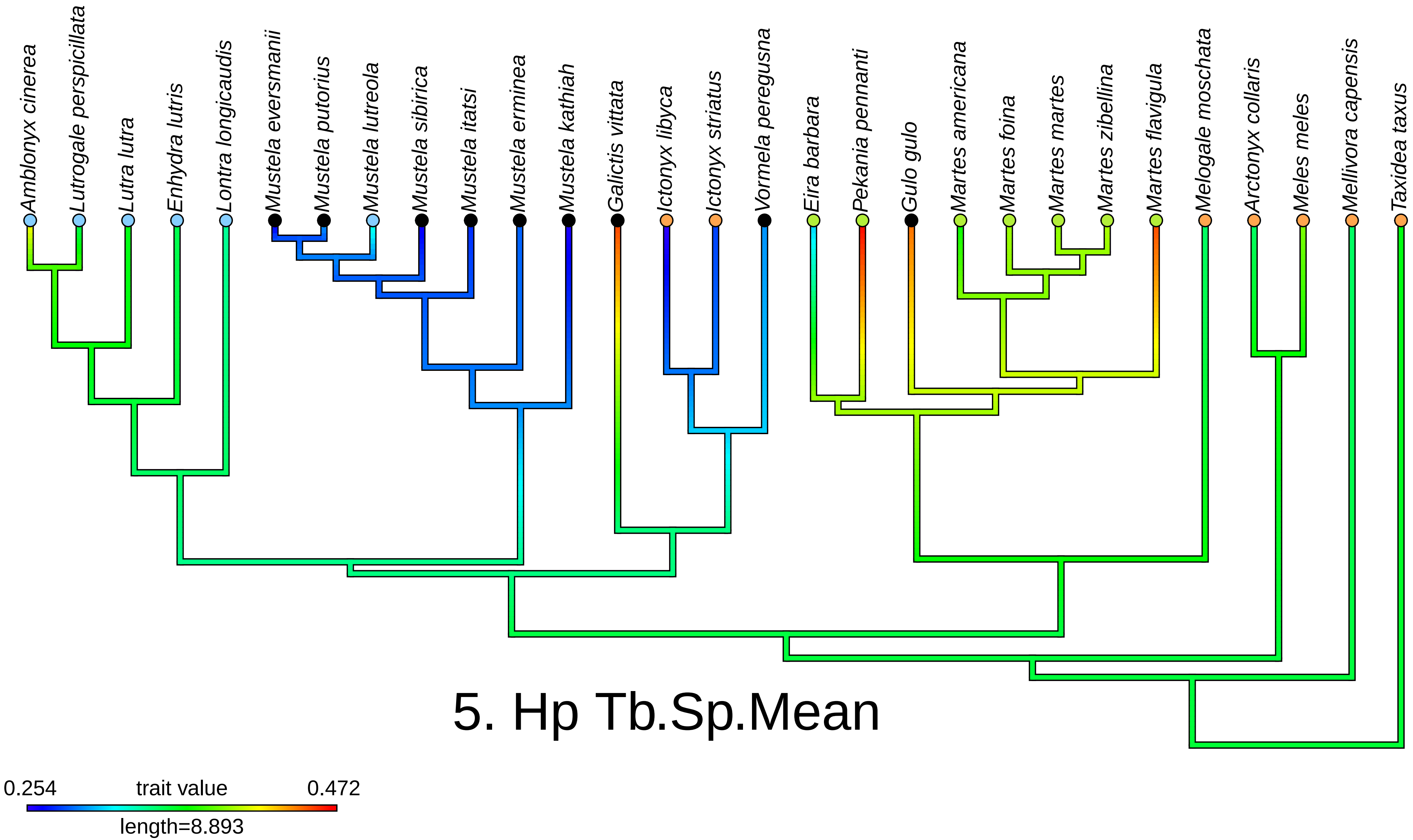
Amblonyx cinerea
Lutrogale perspicillata
Lutra lutra
Enhydra lutris
Lontra longicaudis
Mustela eversmanii
Mustela putorius
Mustela lutreola
Mustela sibirica
Mustela itatsi
Mustela erminea
Mustela kathiah
Galictis vittata
Ictonyx libyca
Ictonyx striatus
Vormela peregusna
Eira barbara
Pekania pennanti
Gulo gulo
Martes americana
Martes foina
Martes martes
Martes zibellina
Martes flavigula
Melogale moschata
Arctonyx collaris
Meles meles
Mellivora capensis
Taxidea taxus
5. Hp Tb.Sp.Mean
0.254
trait value
0.472
length=8.893

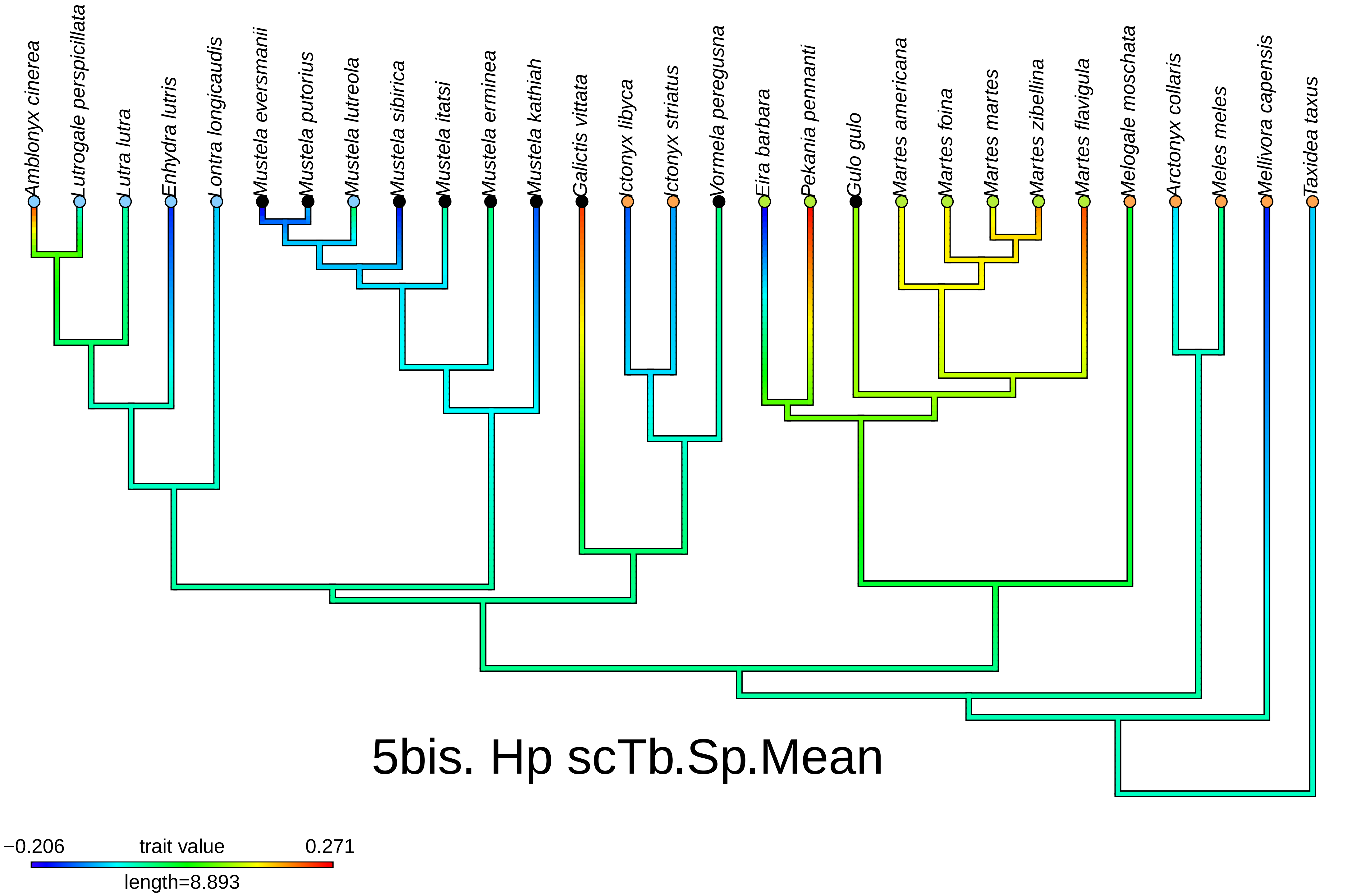

Amblonyx cinerea
Lutrogale perspicillata
Lutra lutra
Enhydra lutris
Lontra longicaudis
Mustela eversmanii
Mustela putorius
Mustela lutreola
Mustela sibirica
Mustela itatsi
Mustela erminea
Mustela kathiah
Galictis vittata
Ictonyx libyca
Ictonyx striatus
Vormela peregusna
Eira barbara
Pekania pennanti
Gulo gulo
Martes americana
Martes foina
Martes martes
Martes zibellina
Martes flavigula
Melogale moschata
Arctonyx collaris
Meles meles
Mellivora capensis
Taxidea taxus
5bis. Hp scTb.Sp.Mean
−0.206
trait value
0.271
length=8.893

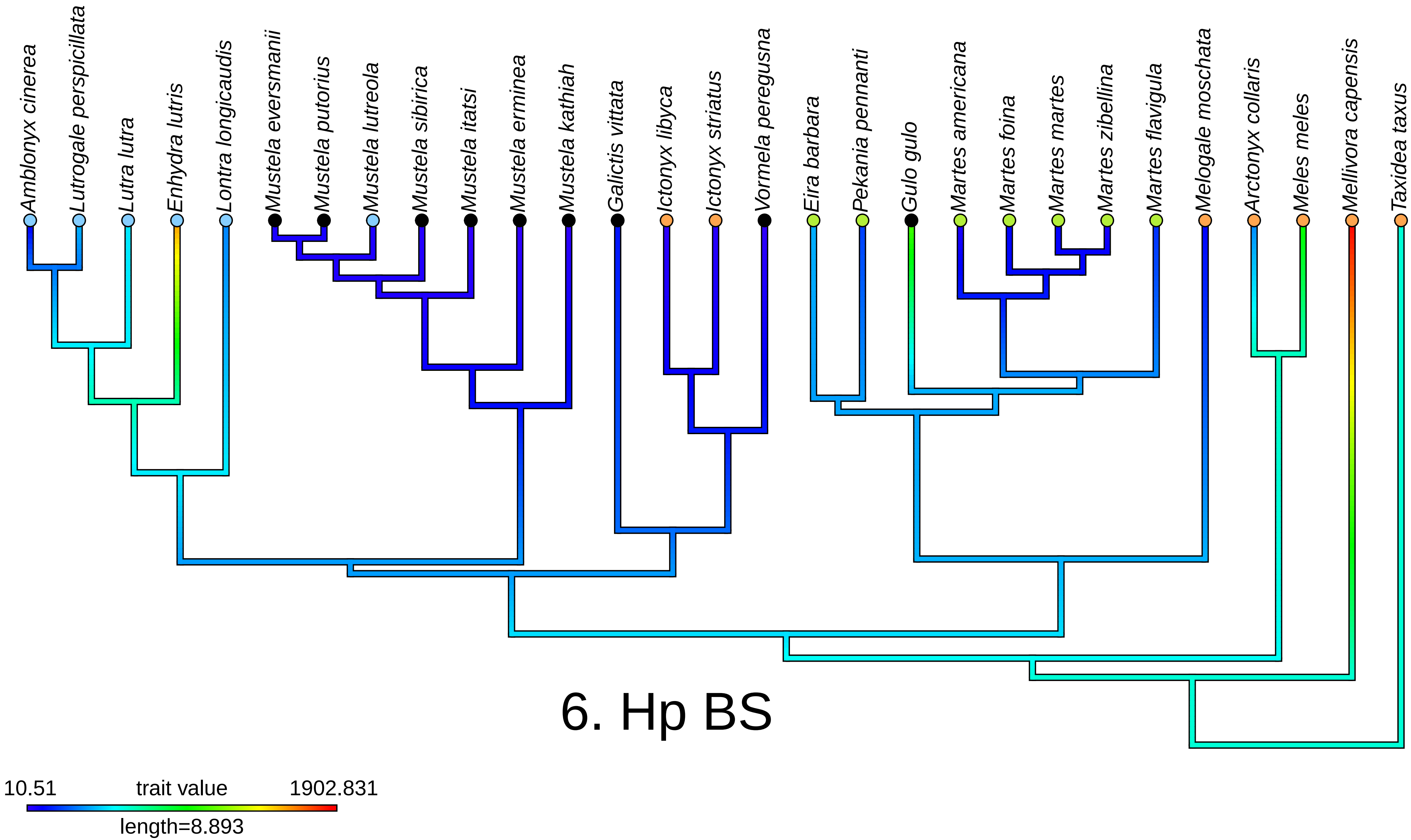
Amblonyx cinerea
Lutrogale perspicillata
Lutra lutra
Enhydra lutris
Lontra longicaudis
Mustela eversmanii
Mustela putorius
Mustela lutreola
Mustela sibirica
Mustela itatsi
Mustela erminea
Mustela kathiah
Galictis vittata
Ictonyx libyca
Ictonyx striatus
Vormela peregusna
Eira barbara
Pekania pennanti
Gulo gulo
Martes americana
Martes foina
Martes martes
Martes zibellina
Martes flavigula
Melogale moschata
Arctonyx collaris
Meles meles
Mellivora capensis
Taxidea taxus
10.51
trait value
1902.831
length=8.893

6. Hp BS

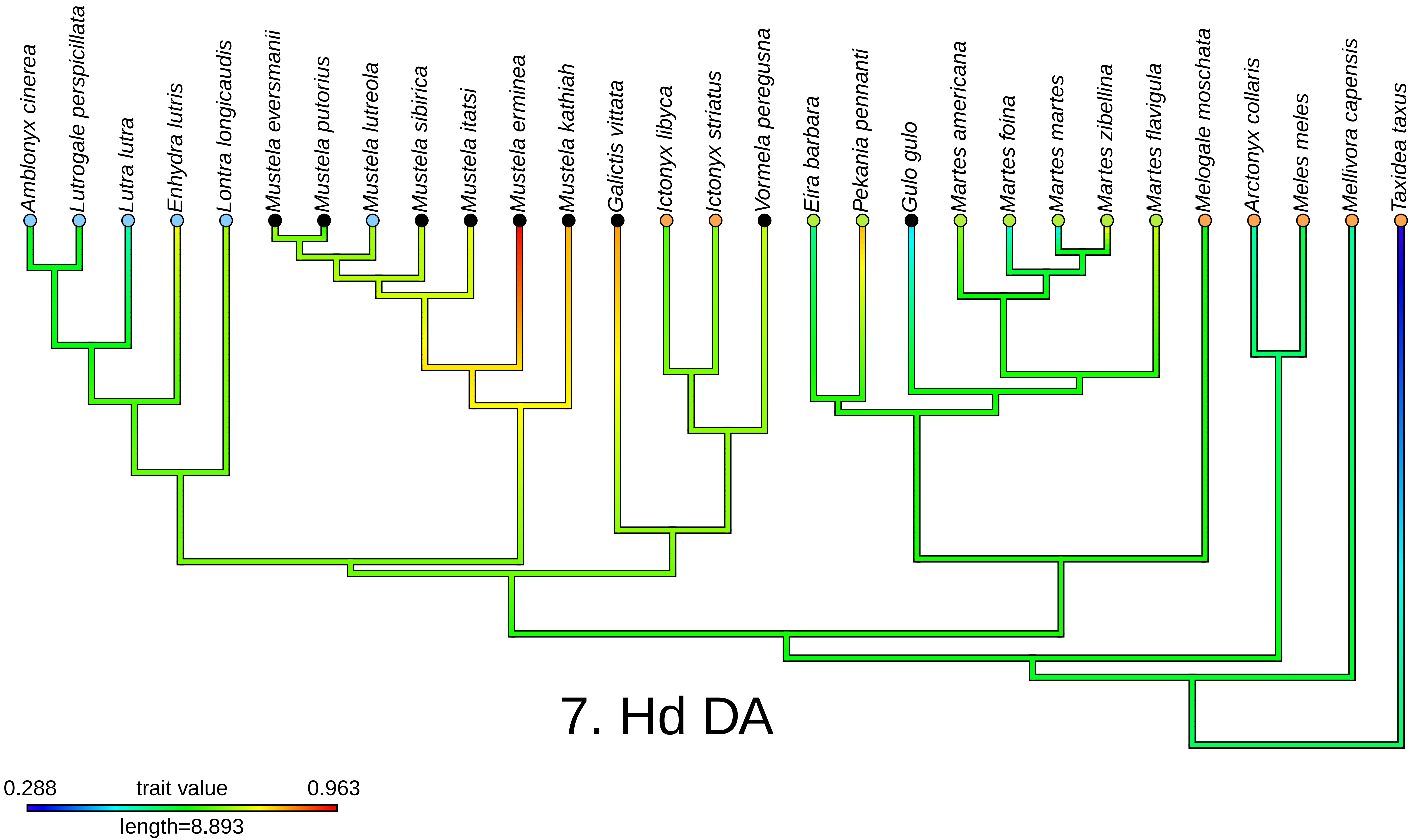

Amblonyx cinerea
Lutrogale perspicillata
Lutra lutra
Enhydra lutris
Lontra longicaudis
Mustela eversmanii
Mustela putorius
Mustela lutreola
Mustela sibirica
Mustela itatsi
Mustela erminea
Mustela kathiah
Galictis vittata
Ictonyx libyca
Ictonyx striatus
Vormela peregusna
Eira barbara
Pekania pennanti
Gulo gulo
Martes americana
Martes foina
Martes martes
Martes zibellina
Martes flavigula
Melogale moschata
Arctonyx collaris
Meles meles
Mellivora capensis
Taxidea taxus
0.288
trait value
0.963
length=8.893

7. Hd DA

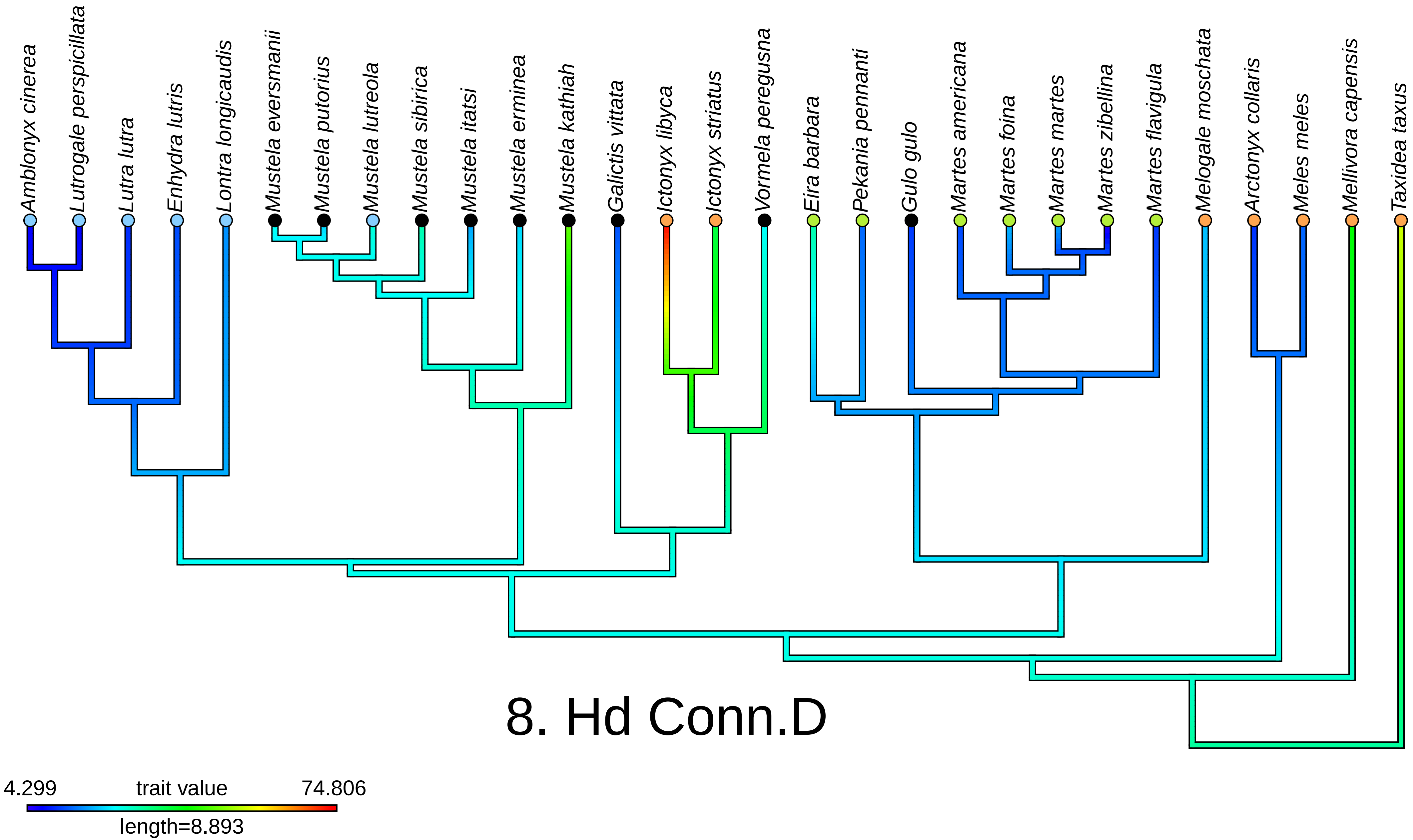
Amblonyx cinerea
Lutrogale perspicillata
Lutra lutra
Enhydra lutris
Lontra longicaudis
Mustela eversmanii
Mustela putorius
Mustela lutreola
Mustela sibirica
Mustela itatsi
Mustela erminea
Mustela kathiah
Galictis vittata
Ictonyx libyca
Ictonyx striatus
Vormela peregusna
Eira barbara
Pekania pennanti
Gulo gulo
Martes americana
Martes foina
Martes martes
Martes zibellina
Martes flavigula
Melogale moschata
Arctonyx collaris
Meles meles
Mellivora capensis
Taxidea taxus
4.299
trait value
74.806
length=8.893

8. Hd Conn.D

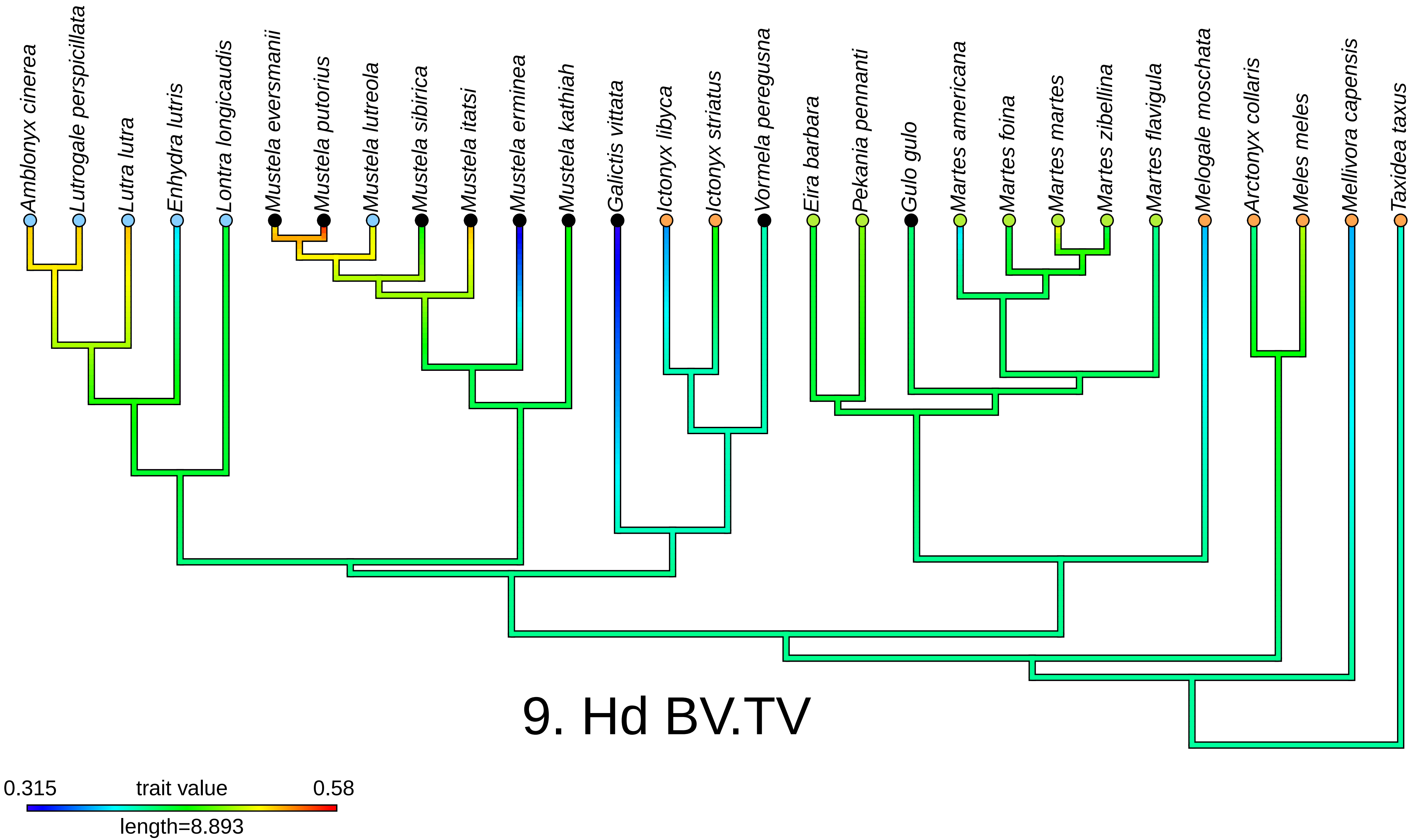

Amblonyx cinerea
Lutrogale perspicillata
Lutra lutra
Enhydra lutris
Lontra longicaudis
Mustela eversmanii
Mustela putorius
Mustela lutreola
Mustela sibirica
Mustela itatsi
Mustela erminea
Mustela kathiah
Galictis vittata
Ictonyx libyca
Ictonyx striatus
Vormela peregusna
Eira barbara
Pekania pennanti
Gulo gulo
Martes americana
Martes foina
Martes martes
Martes zibellina
Martes flavigula
Melogale moschata
Arctonyx collaris
Meles meles
Mellivora capensis
Taxidea taxus
0.315
trait value
0.58
length=8.893

9. Hd BV.TV

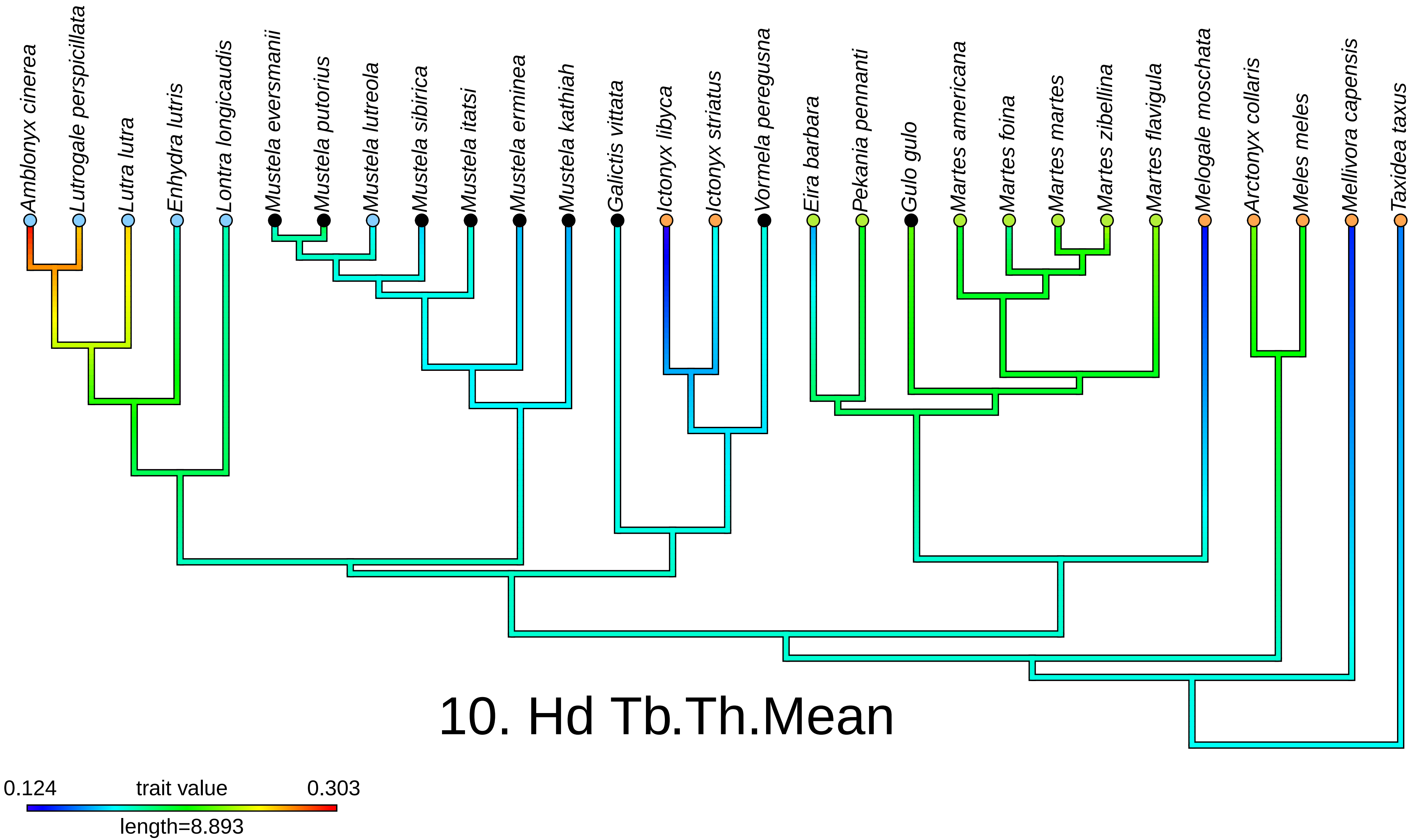
Amblonyx cinerea
Lutrogale perspicillata
Lutra lutra
Enhydra lutris
Lontra longicaudis
Mustela eversmanii
Mustela putorius
Mustela lutreola
Mustela sibirica
Mustela itatsi
Mustela erminea
Mustela kathiah
Galictis vittata
Ictonyx libyca
Ictonyx striatus
Vormela peregusna
Eira barbara
Pekania pennanti
Gulo gulo
Martes americana
Martes foina
Martes martes
Martes zibellina
Martes flavigula
Melogale moschata
Arctonyx collaris
Meles meles
Mellivora capensis
Taxidea taxus
10. Hd Tb.Th.Mean
0.124
trait value
0.303
length=8.893

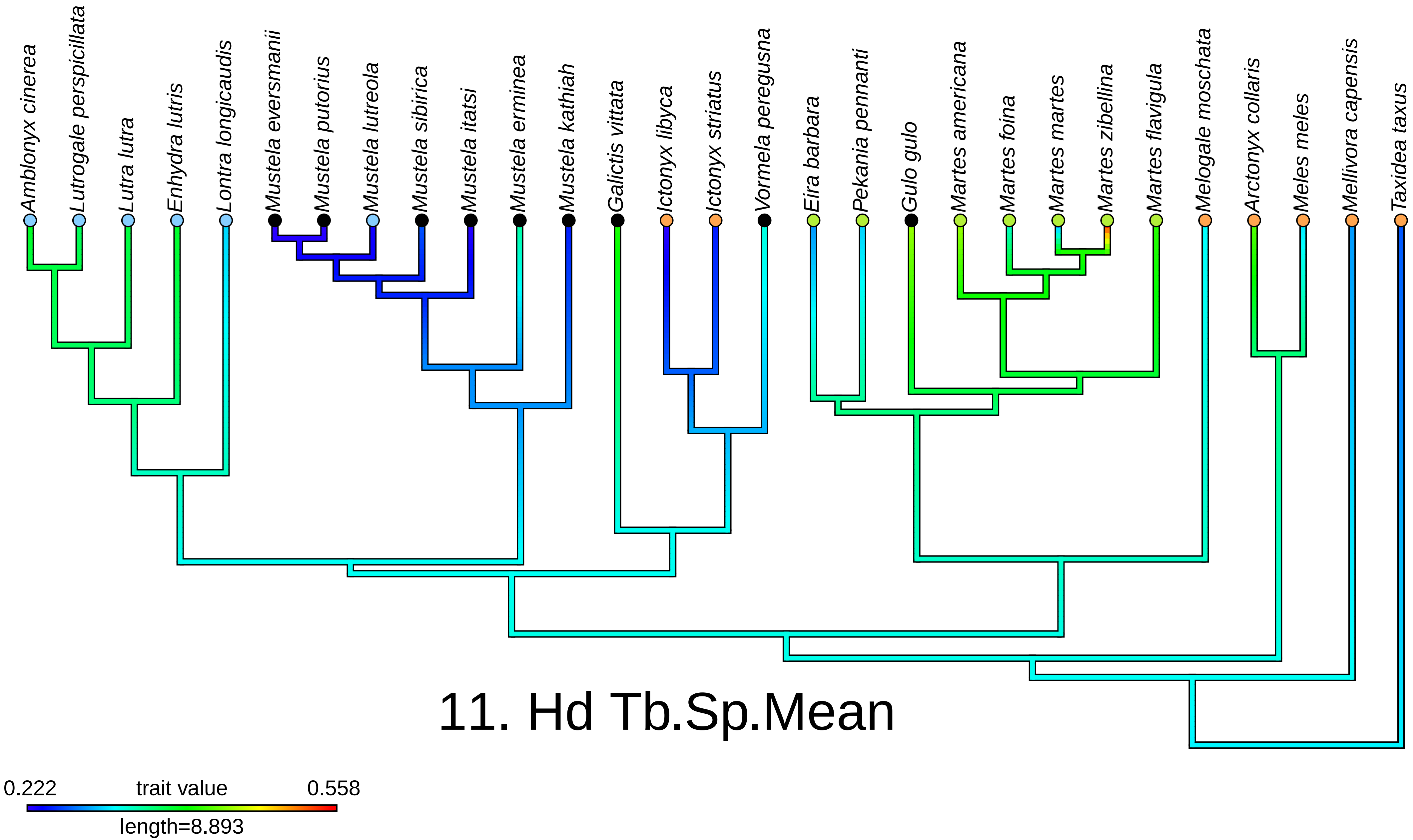

11. Hd Tb.Sp.Mean

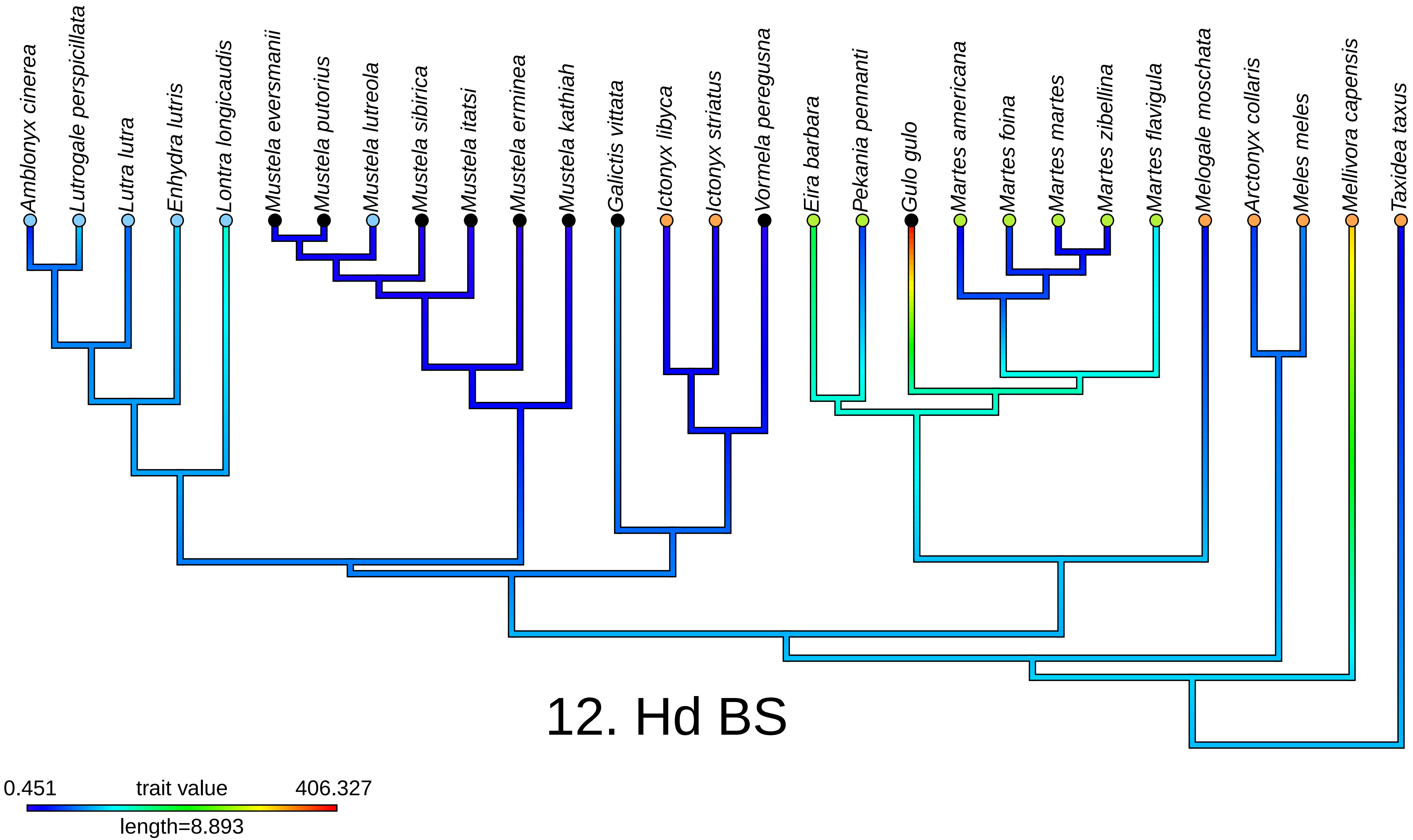
Amblonyx cinerea
Lutrogale perspicillata
Lutra lutra
Enhydra lutris
Lontra longicaudis
Mustela eversmanii
Mustela putorius
Mustela lutreola
Mustela sibirica
Mustela itatsi
Mustela erminea
Mustela kathiah
Galictis vittata
Ictonyx libyca
Ictonyx striatus
Vormela peregusna
Eira barbara
Pekania pennanti
Gulo gulo
Martes americana
Martes foina
Martes martes
Martes zibellina
Martes flavigula
Melogale moschata
Arctonyx collaris
Meles meles
Mellivora capensis
Taxidea taxus
12. Hd BS
0.451
trait value
406.327
length=8.893

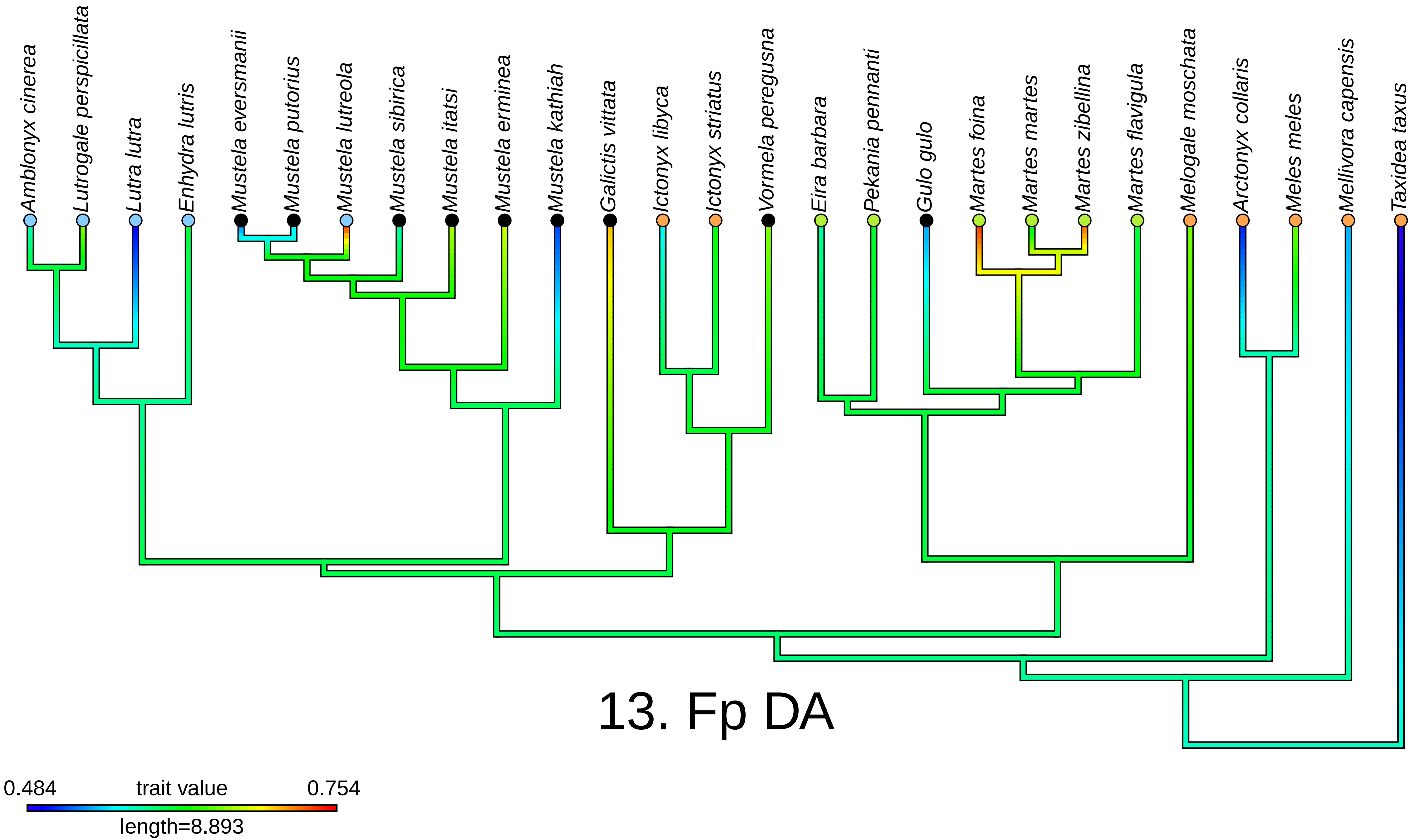
Amblonyx cinerea
Lutrogale perspicillata
Lutra lutra
Enhydra lutris
Mustela eversmanii
Mustela putorius
Mustela lutreola
Mustela sibirica
Mustela itatsi
Mustela erminea
Mustela kathiah
Galictis vittata
Ictonyx libyca
Ictonyx striatus
Vormela peregusna
Eira barbara
Pekania pennanti
Gulo gulo
Martes foina
Martes martes
Martes zibellina
Martes flavigula
Melogale moschata
Arctonyx collaris
Meles meles
Mellivora capensis
Taxidea taxus
0.484
trait value
0.754
length=8.893

13. Fp DA

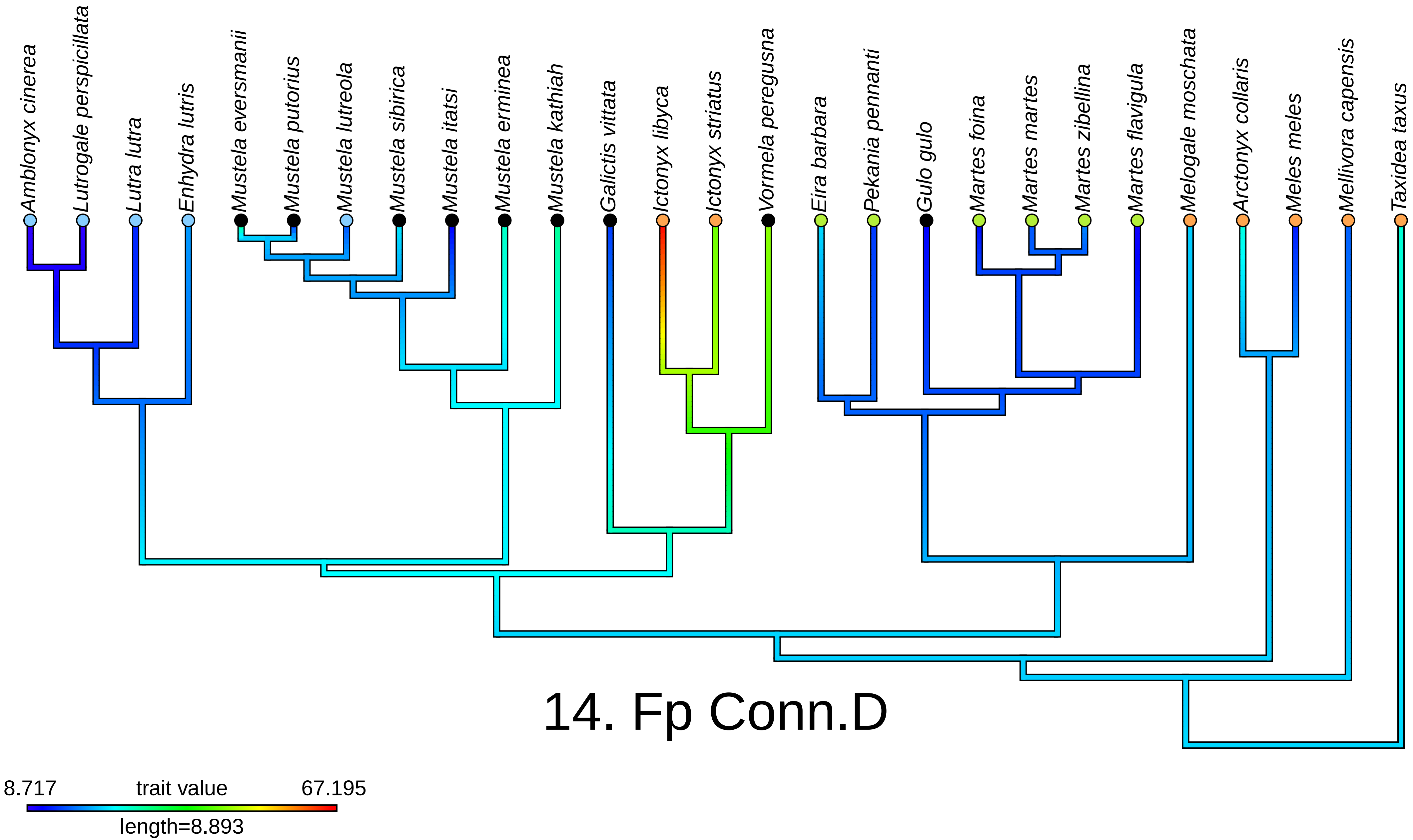
Amblonyx cinerea
Lutrogale perspicillata
Lutra lutra
Enhydra lutris
Mustela eversmanii
Mustela putorius
Mustela lutreola
Mustela sibirica
Mustela itatsi
Mustela erminea
Mustela kathiah
Galictis vittata
Ictonyx libyca
Ictonyx striatus
Vormela peregusna
Eira barbara
Pekania pennanti
Gulo gulo
Martes foina
Martes martes
Martes zibellina
Martes flavigula
Melogale moschata
Arctonyx collaris
Meles meles
Mellivora capensis
Taxidea taxus
14. Fp Conn.D
8.717
trait value
67.195
length=8.893

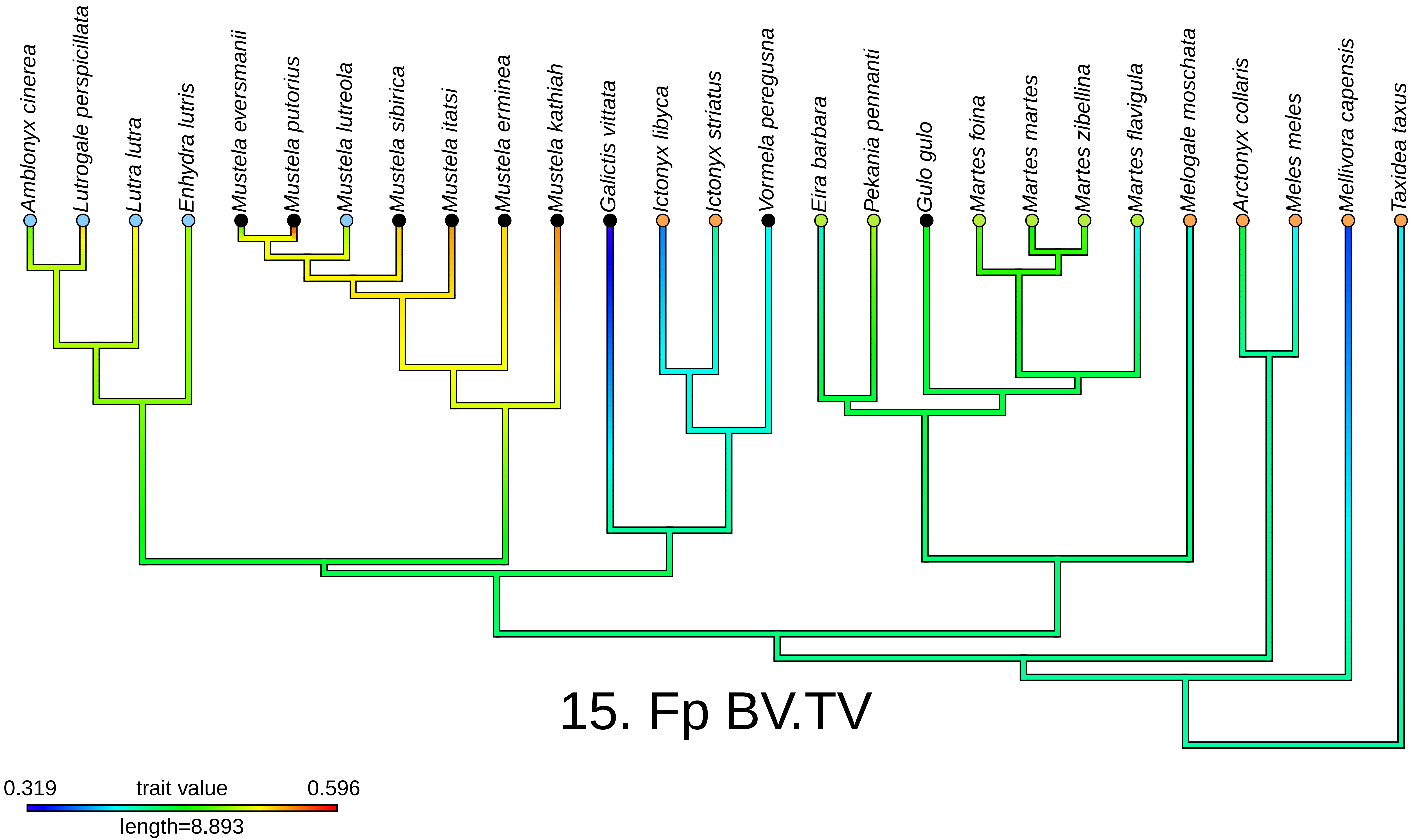

Amblonyx cinerea
Lutrogale perspicillata
Lutra lutra
Enhydra lutris
Mustela eversmanii
Mustela putorius
Mustela lutreola
Mustela sibirica
Mustela itatsi
Mustela erminea
Mustela kathiah
Galictis vittata
Ictonyx libyca
Ictonyx striatus
Vormela peregusna
Eira barbara
Pekania pennanti
Gulo gulo
Martes foina
Martes martes
Martes zibellina
Martes flavigula
Melogale moschata
Arctonyx collaris
Meles meles
Mellivora capensis
Taxidea taxus
15. Fp BV.TV
0.319
trait value
0.596
length=8.893

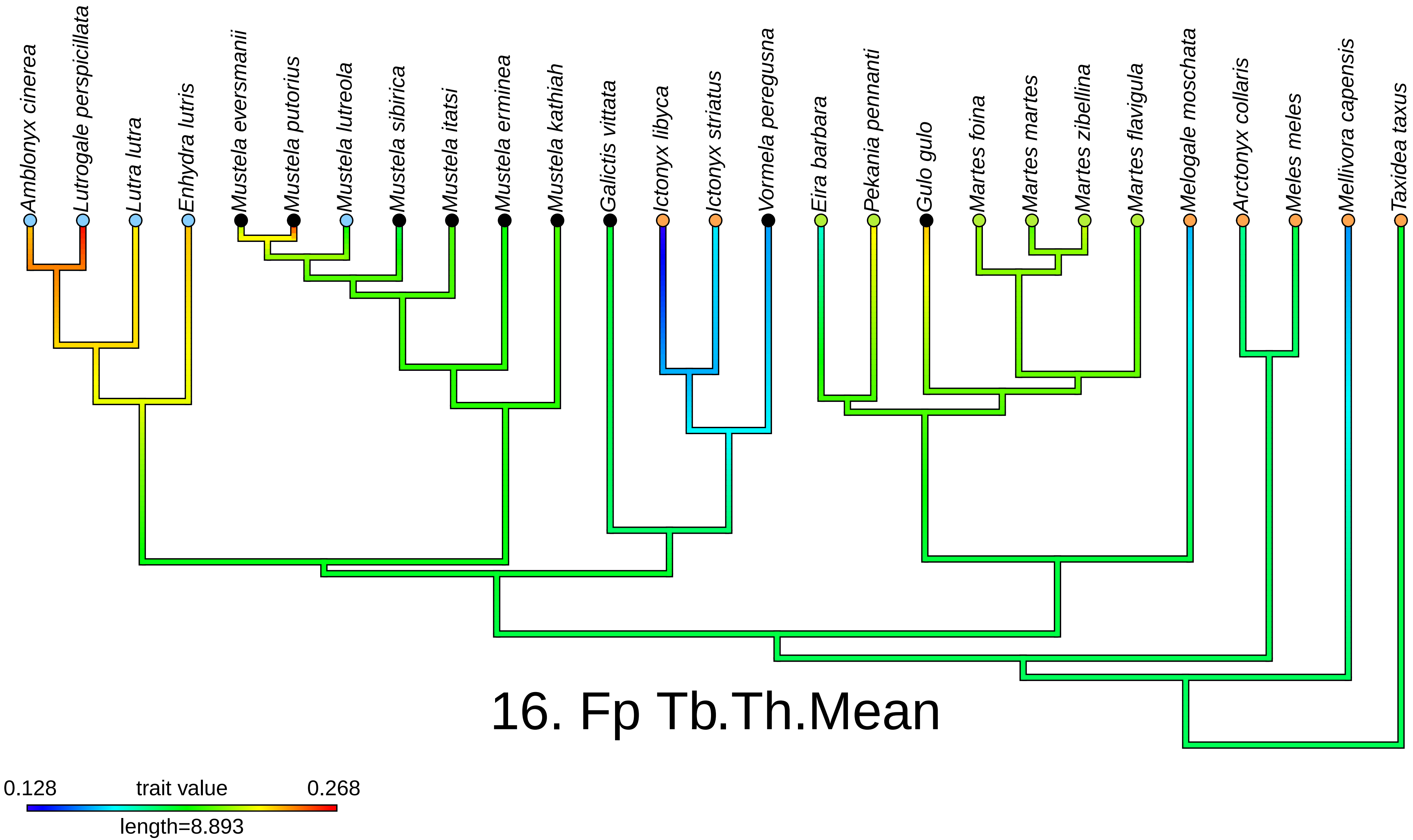
Amblonyx cinerea
Lutrogale perspicillata
Lutra lutra
Enhydra lutris
Mustela eversmanii
Mustela putorius
Mustela lutreola
Mustela sibirica
Mustela itatsi
Mustela erminea
Mustela kathiah
Galictis vittata
Ictonyx libyca
Ictonyx striatus
Vormela peregusna
Eira barbara
Pekania pennanti
Gulo gulo
Martes foina
Martes martes
Martes zibellina
Martes flavigula
Melogale moschata
Arctonyx collaris
Meles meles
Mellivora capensis
Taxidea taxus
16. Fp Tb.Th.Mean
0.128
trait value
0.268
length=8.893

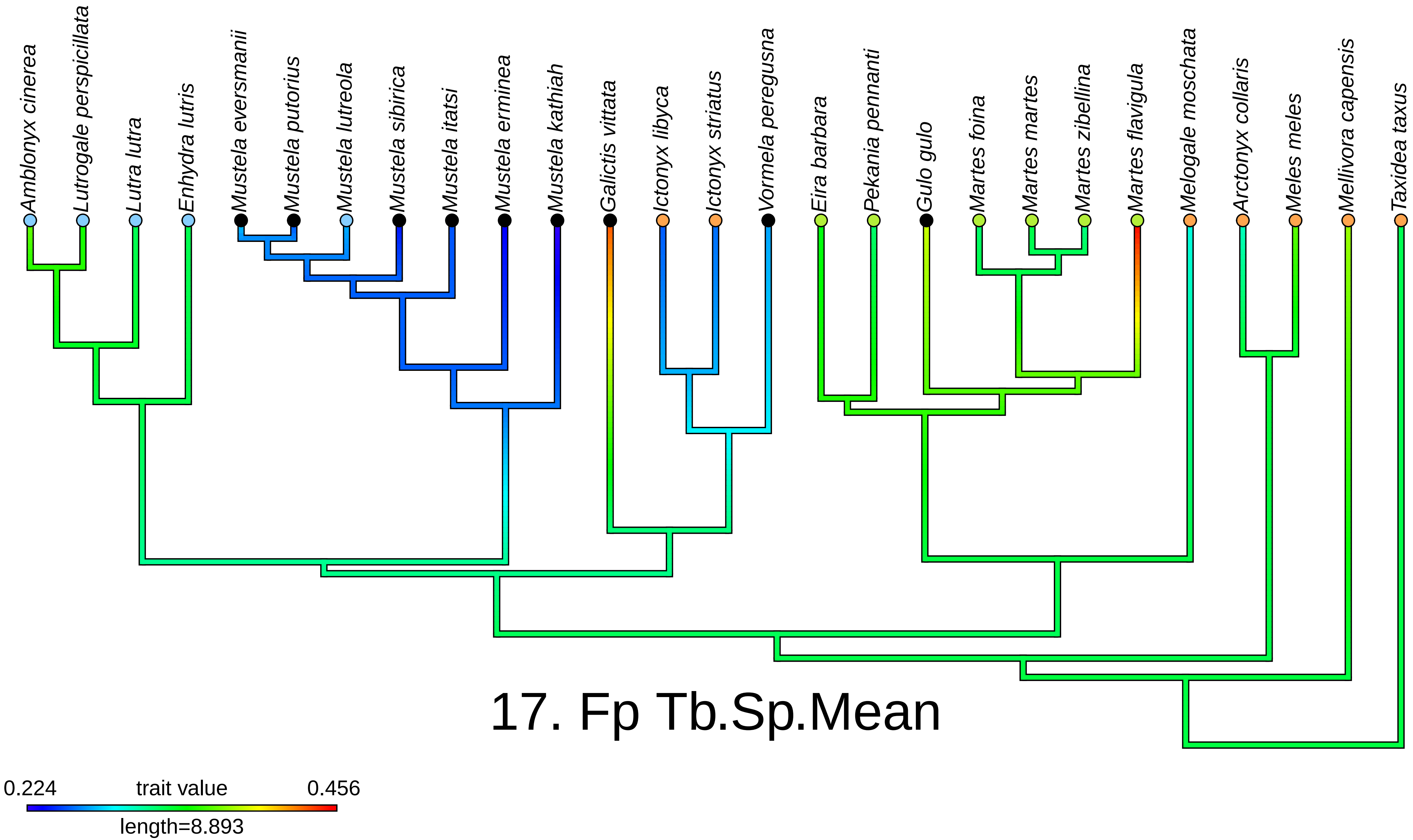

17. Fp Tb.Sp.Mean

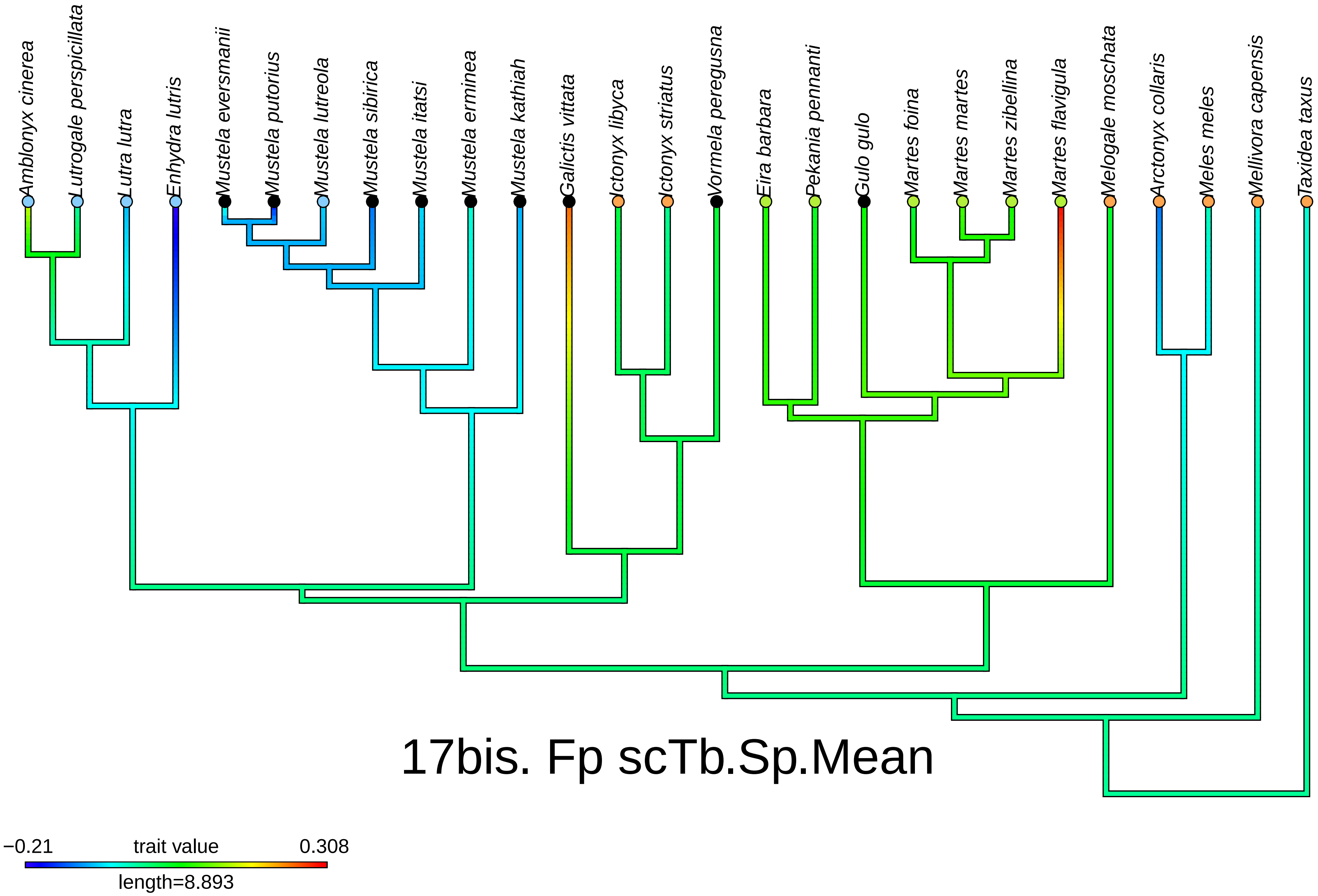
Amblonyx cinerea
Lutrogale perspicillata
Lutra lutra
Enhydra lutris
Mustela eversmanii
Mustela putorius
Mustela lutreola
Mustela sibirica
Mustela itatsi
Mustela erminea
Mustela kathiah
Galictis vittata
Ictonyx libyca
Ictonyx striatus
Vormela peregusna
Eira barbara
Pekania pennanti
Gulo gulo
Martes foina
Martes martes
Martes zibellina
Martes flavigula
Melogale moschata
Arctonyx collaris
Meles meles
Mellivora capensis
Taxidea taxus
17bis. Fp scTb.Sp.Mean
−0.21
trait value
0.308
length=8.893

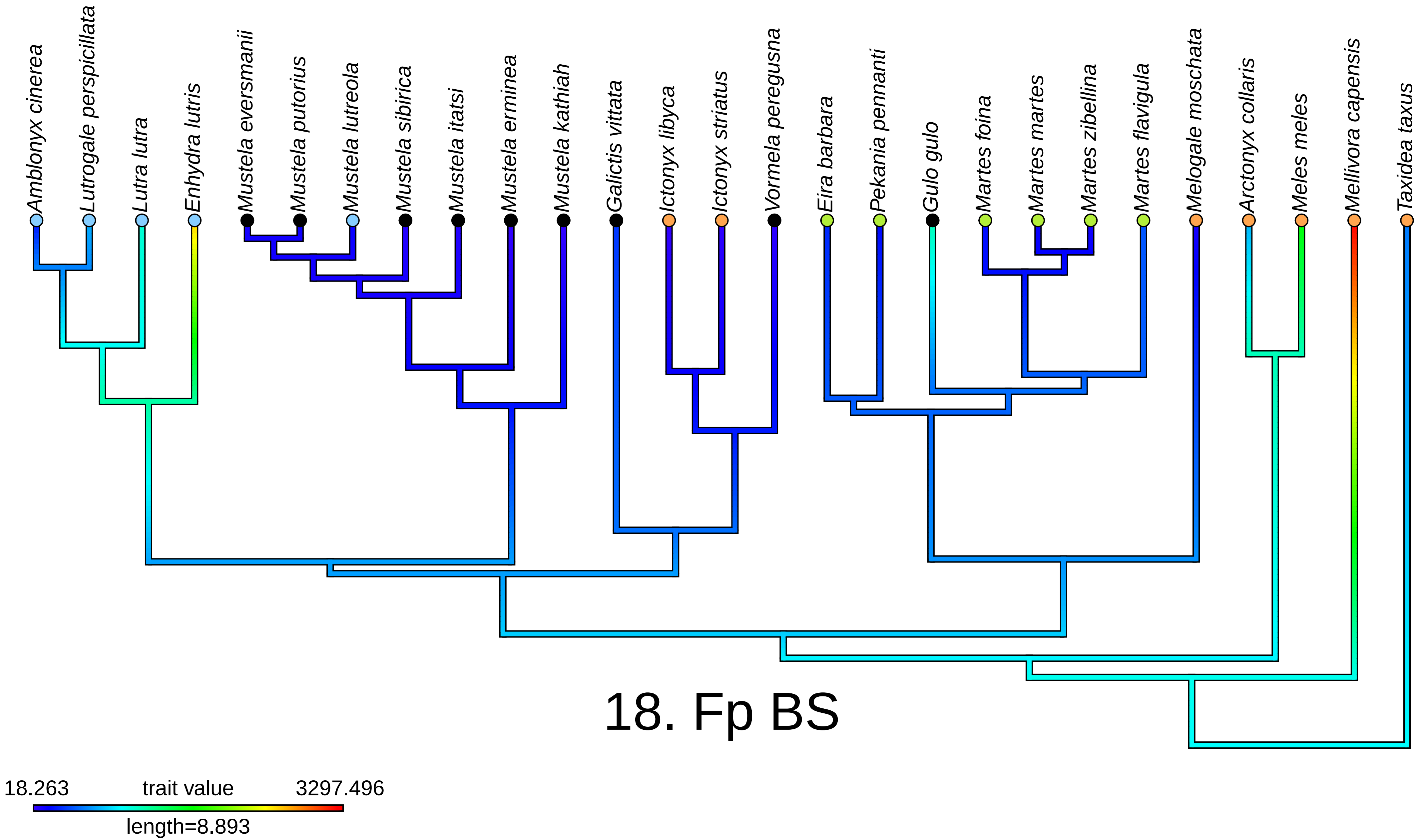

Amblonyx cinerea
Lutrogale perspicillata
Lutra lutra
Enhydra lutris
Mustela eversmanii
Mustela putorius
Mustela lutreola
Mustela sibirica
Mustela itatsi
Mustela erminea
Mustela kathiah
Galictis vittata
Ictonyx libyca
Ictonyx striatus
Vormela peregusna
Eira barbara
Pekania pennanti
Gulo gulo
Martes foina
Martes martes
Martes zibellina
Martes flavigula
Melogale moschata
Arctonyx collaris
Meles meles
Mellivora capensis
Taxidea taxus
18.263
trait value
3297.496
length=8.893

18. Fp BS

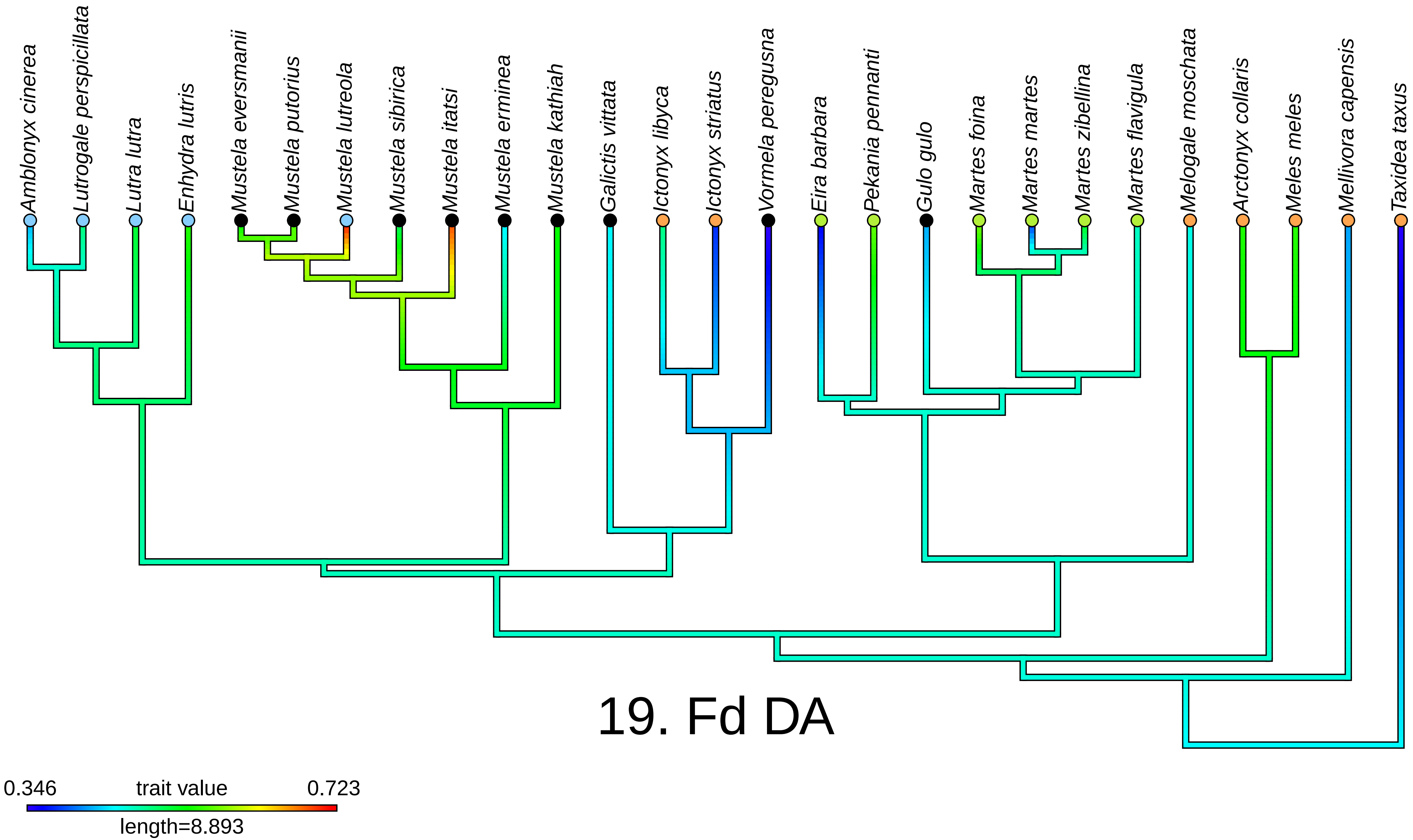
Amblonyx cinerea
Lutrogale perspicillata
Lutra lutra
Enhydra lutris
Mustela eversmanii
Mustela putorius
Mustela lutreola
Mustela sibirica
Mustela itatsi
Mustela erminea
Mustela kathiah
Galictis vittata
Ictonyx libyca
Ictonyx striatus
Vormela peregusna
Eira barbara
Pekania pennanti
Gulo gulo
Martes foina
Martes martes
Martes zibellina
Martes flavigula
Melogale moschata
Arctonyx collaris
Meles meles
Mellivora capensis
Taxidea taxus
0.346
trait value
0.723
length=8.893

19. Fd DA

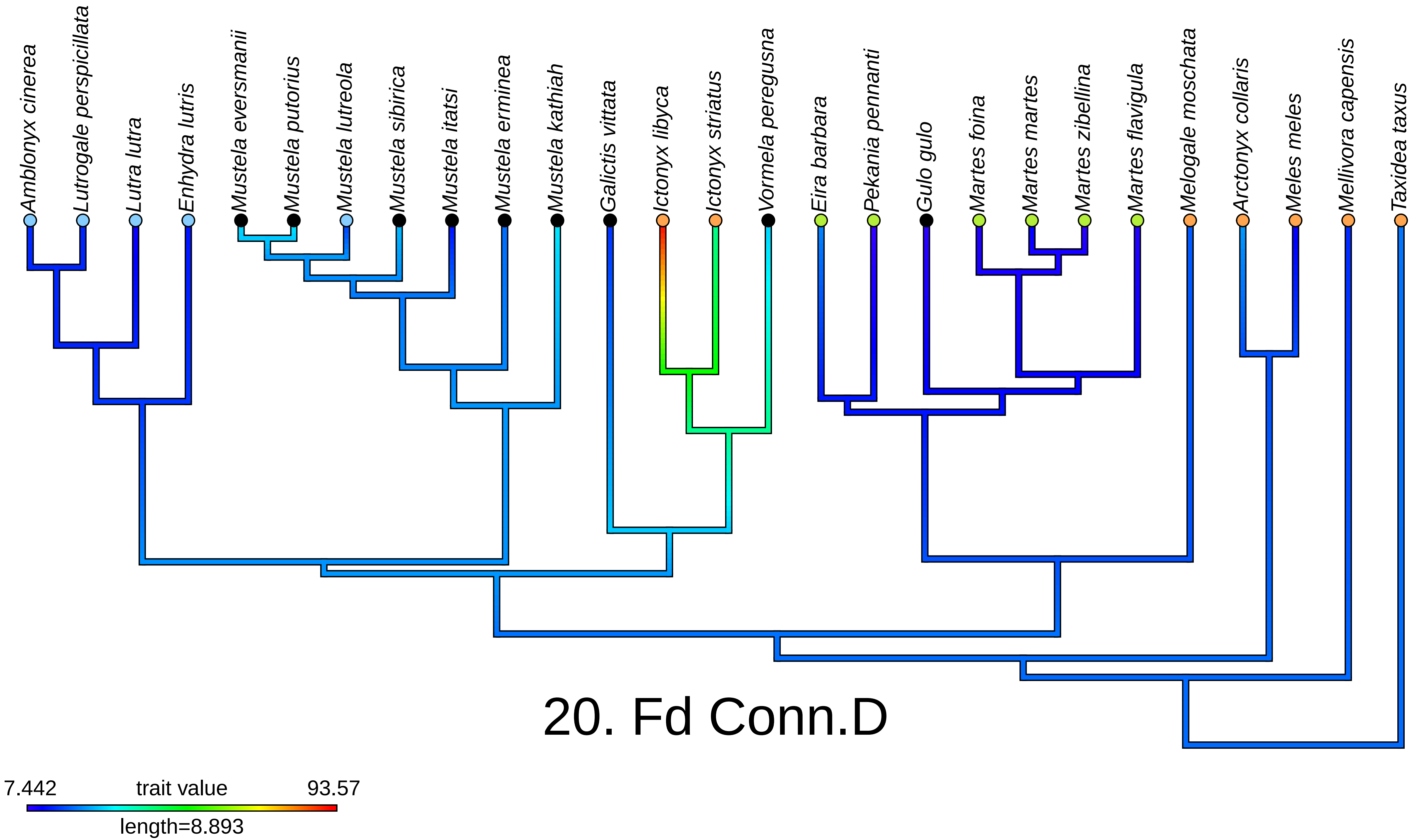

Amblonyx cinerea
Lutrogale perspicillata
Lutra lutra
Enhydra lutris
Mustela eversmanii
Mustela putorius
Mustela lutreola
Mustela sibirica
Mustela itatsi
Mustela erminea
Mustela kathiah
Galictis vittata
Ictonyx libyca
Ictonyx striatus
Vormela peregusna
Eira barbara
Pekania pennanti
Gulo gulo
Martes foina
Martes martes
Martes zibellina
Martes flavigula
Melogale moschata
Arctonyx collaris
Meles meles
Mellivora capensis
Taxidea taxus
20. Fd Conn.D
7.442
trait value
93.57
length=8.893

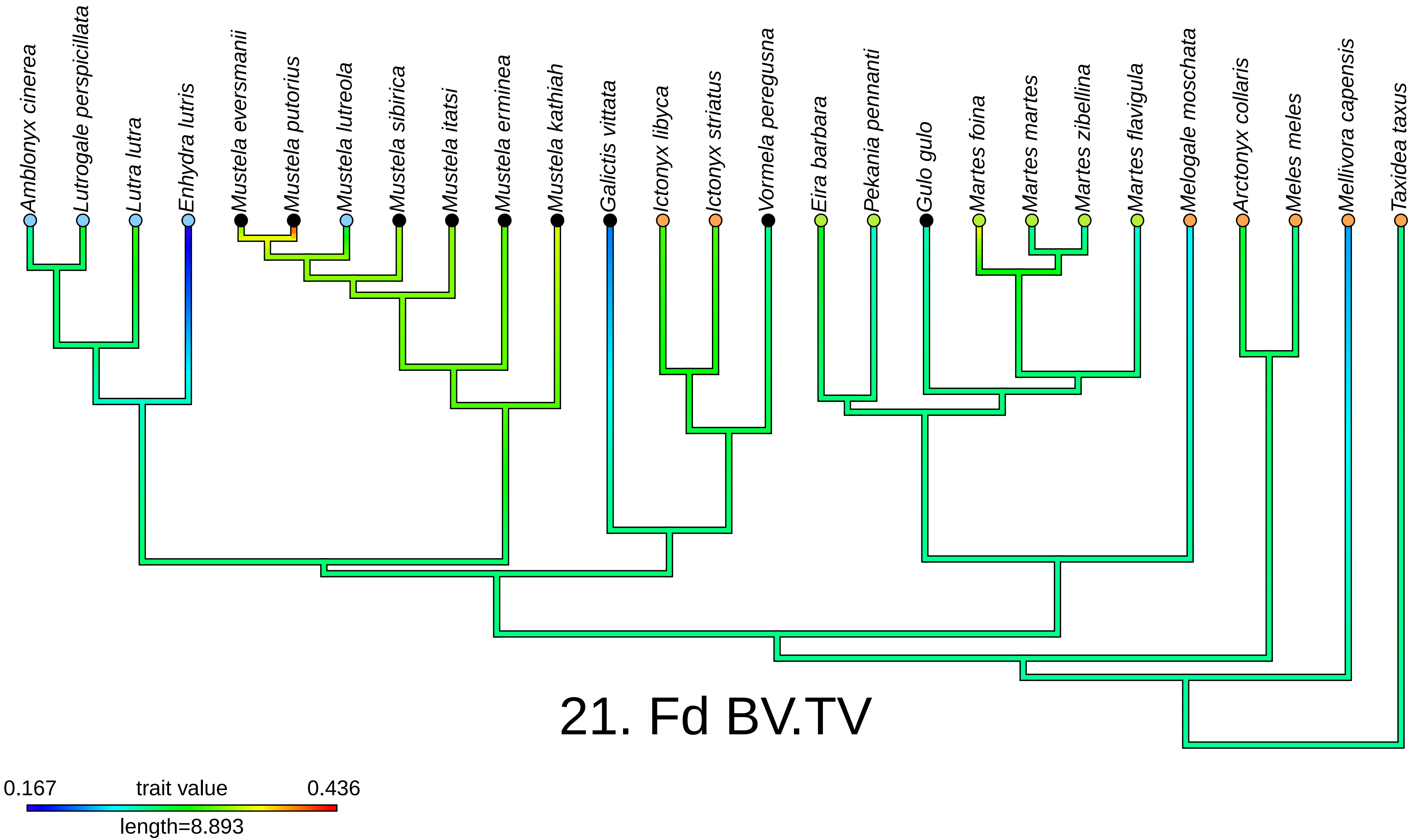
Amblonyx cinerea
Lutrogale perspicillata
Lutra lutra
Enhydra lutris
Mustela eversmanii
Mustela putorius
Mustela lutreola
Mustela sibirica
Mustela itatsi
Mustela erminea
Mustela kathiah
Galictis vittata
Ictonyx libyca
Ictonyx striatus
Vormela peregusna
Eira barbara
Pekania pennanti
Gulo gulo
Martes foina
Martes martes
Martes zibellina
Martes flavigula
Melogale moschata
Arctonyx collaris
Meles meles
Mellivora capensis
Taxidea taxus
21. Fd BV.TV
0.167
trait value
0.436
length=8.893

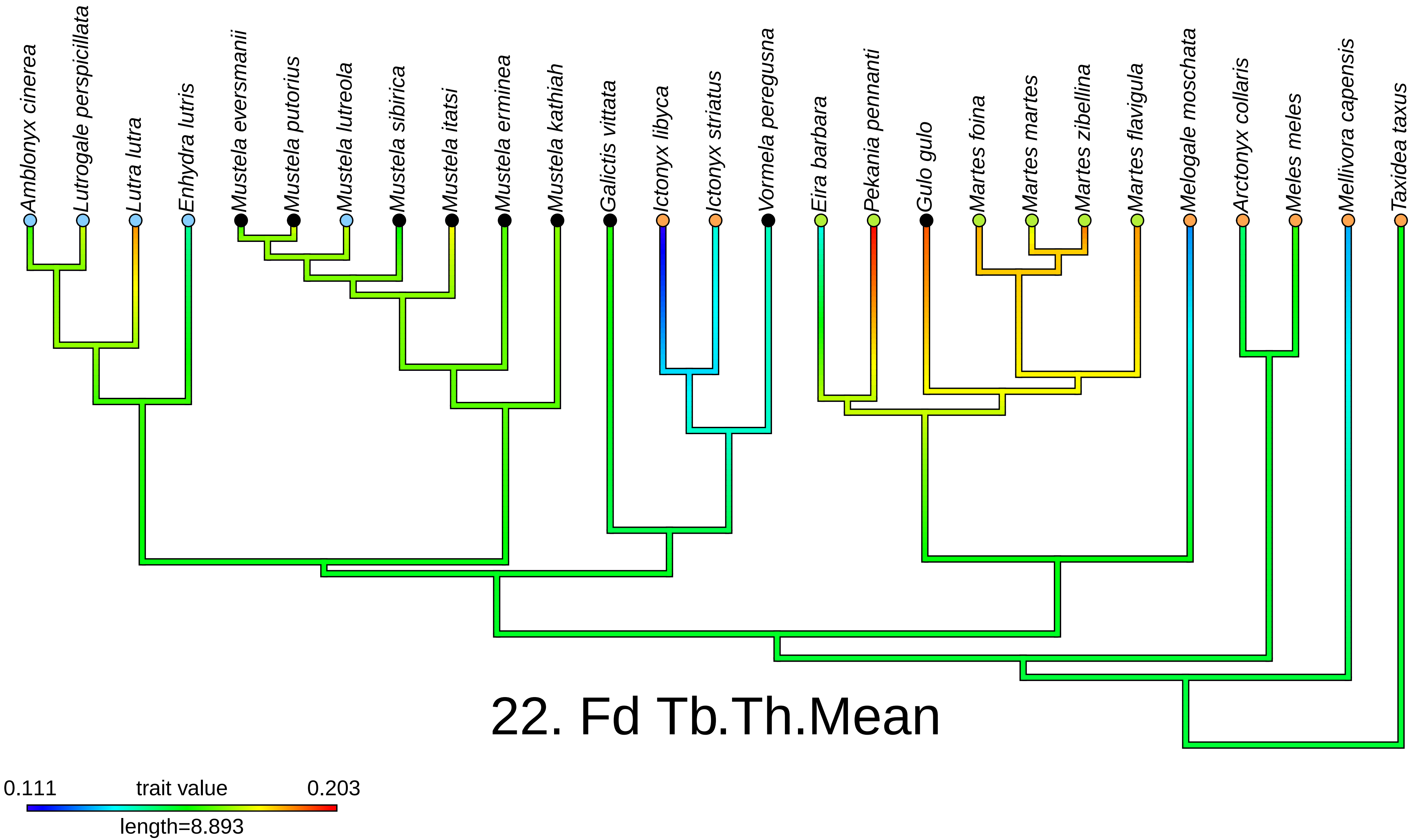
Amblonyx cinerea
Lutrogale perspicillata
Lutra lutra
Enhydra lutris
Mustela eversmanii
Mustela putorius
Mustela lutreola
Mustela sibirica
Mustela itatsi
Mustela erminea
Mustela kathiah
Galictis vittata
Ictonyx libyca
Ictonyx striatus
Vormela peregusna
Eira barbara
Pekania pennanti
Gulo gulo
Martes foina
Martes martes
Martes zibellina
Martes flavigula
Melogale moschata
Arctonyx collaris
Meles meles
Mellivora capensis
Taxidea taxus
22. Fd Tb.Th.Mean
0.111
trait value
0.203
length=8.893

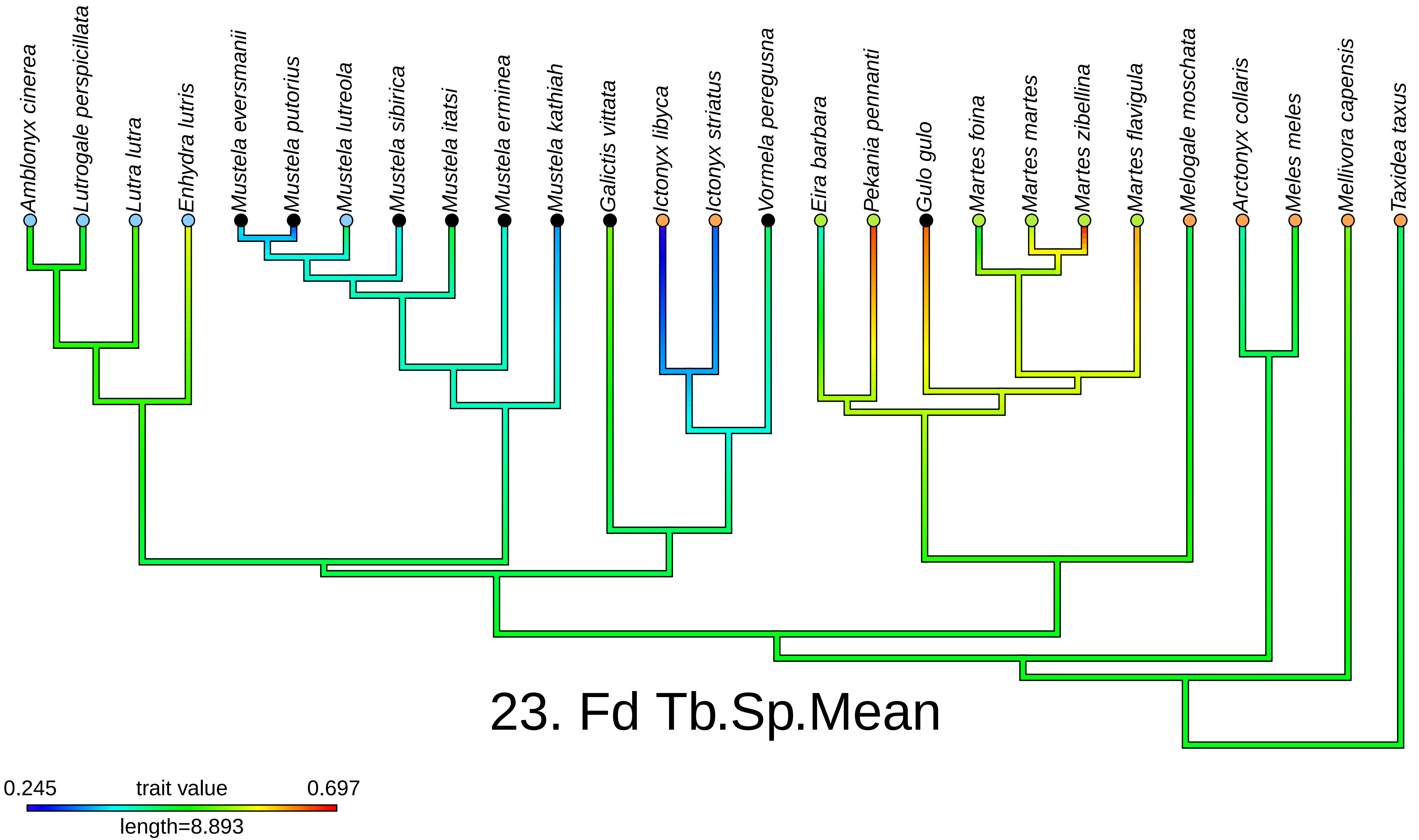

Amblonyx cinerea
Lutrogale perspicillata
Lutra lutra
Enhydra lutris
Mustela eversmanii
Mustela putorius
Mustela lutreola
Mustela sibirica
Mustela itatsi
Mustela erminea
Mustela kathiah
Galictis vittata
Ictonyx libyca
Ictonyx striatus
Vormela peregusna
Eira barbara
Pekania pennanti
Gulo gulo
Martes foina
Martes martes
Martes zibellina
Martes flavigula
Melogale moschata
Arctonyx collaris
Meles meles
Mellivora capensis
Taxidea taxus
23. Fd Tb.Sp.Mean
0.245
trait value
0.697
length=8.893

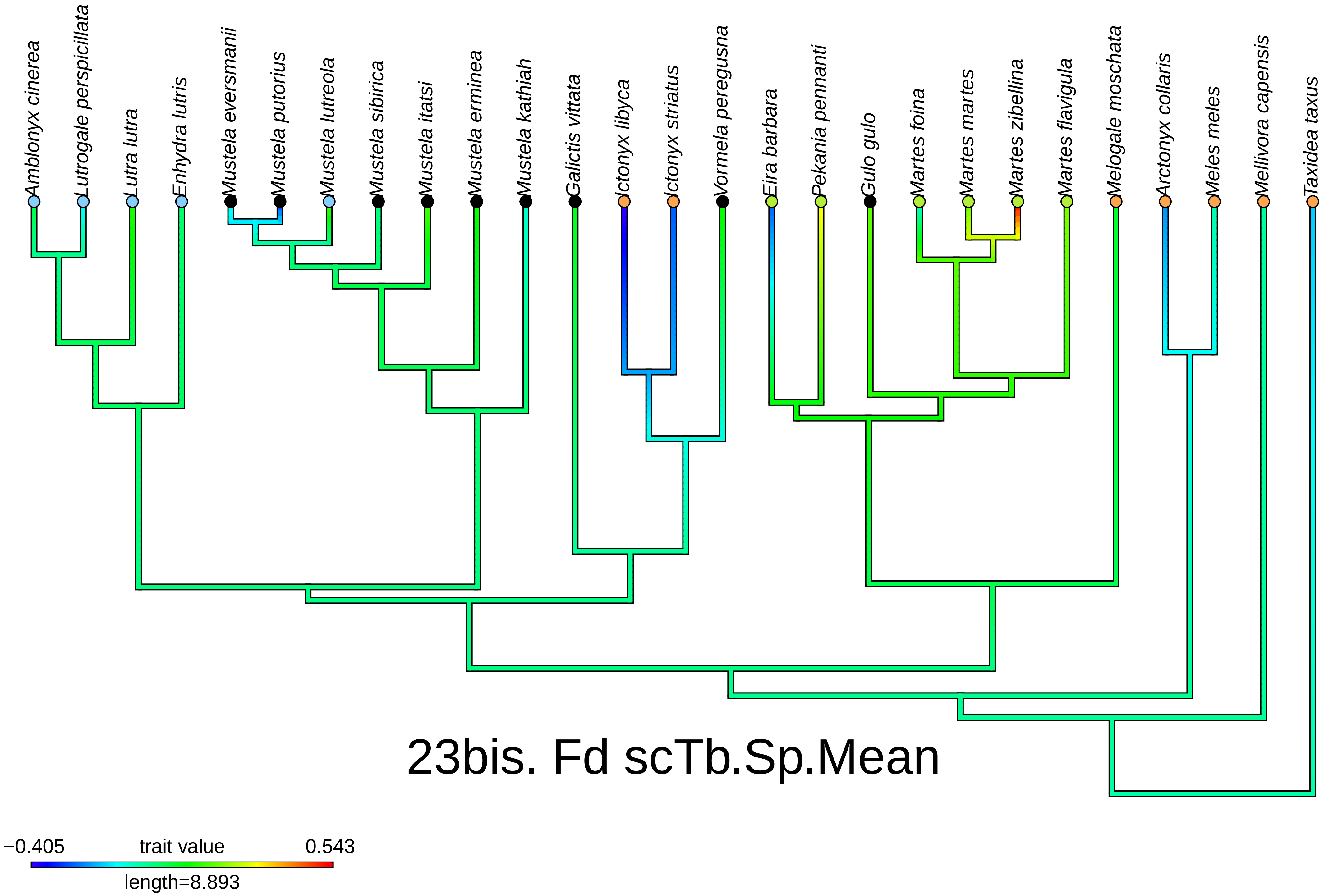

23bis. Fd scTb.Sp.Mean

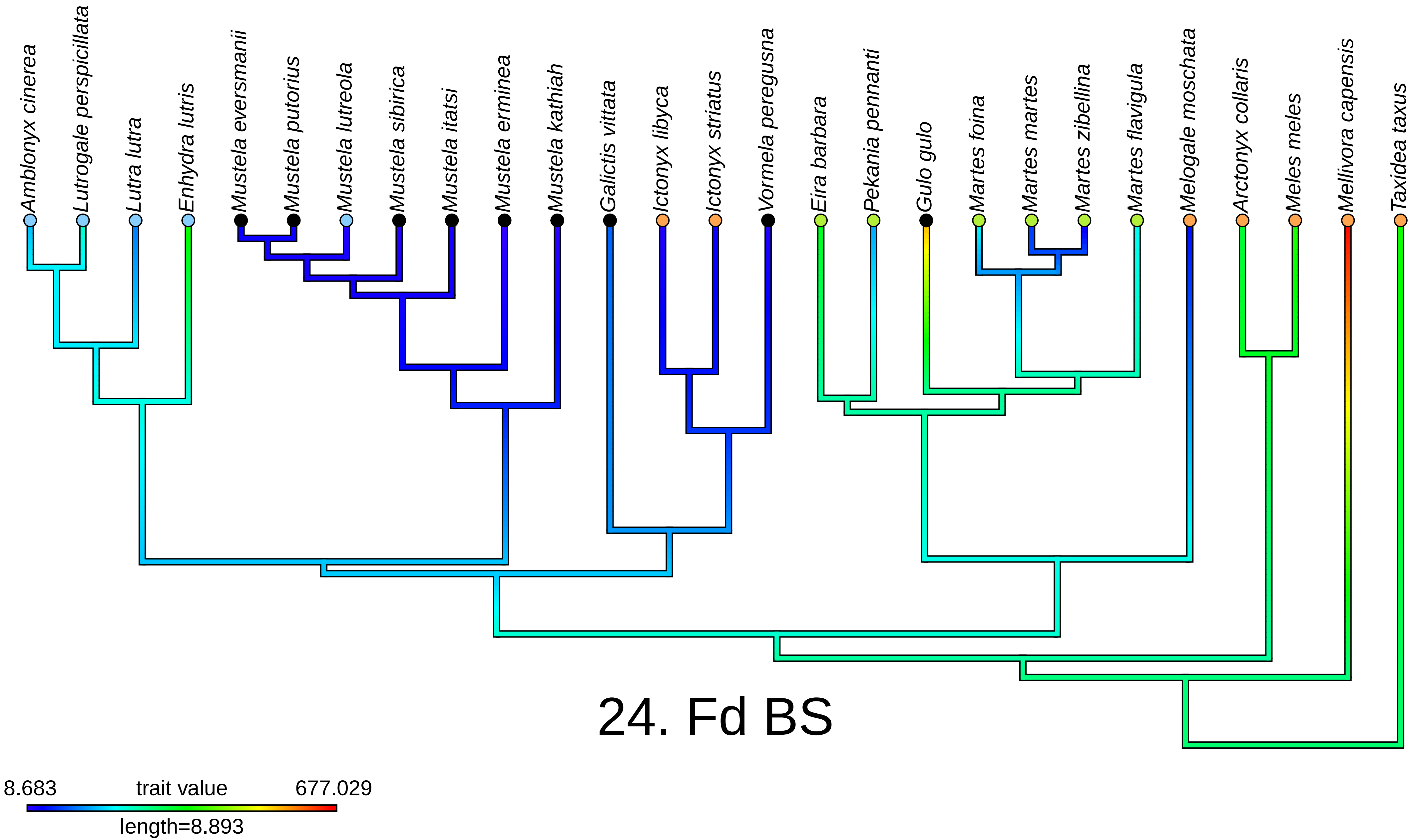
Amblonyx cinerea
Lutrogale perspicillata
Lutra lutra
Enhydra lutris
Mustela eversmanii
Mustela putorius
Mustela lutreola
Mustela sibirica
Mustela itatsi
Mustela erminea
Mustela kathiah
Galictis vittata
Ictonyx libyca
Ictonyx striatus
Vormela peregusna
Eira barbara
Pekania pennanti
Gulo gulo
Martes foina
Martes martes
Martes zibellina
Martes flavigula
Melogale moschata
Arctonyx collaris
Meles meles
Mellivora capensis
Taxidea taxus
8.683
trait value
677.029
length=8.893

24. Fd BS

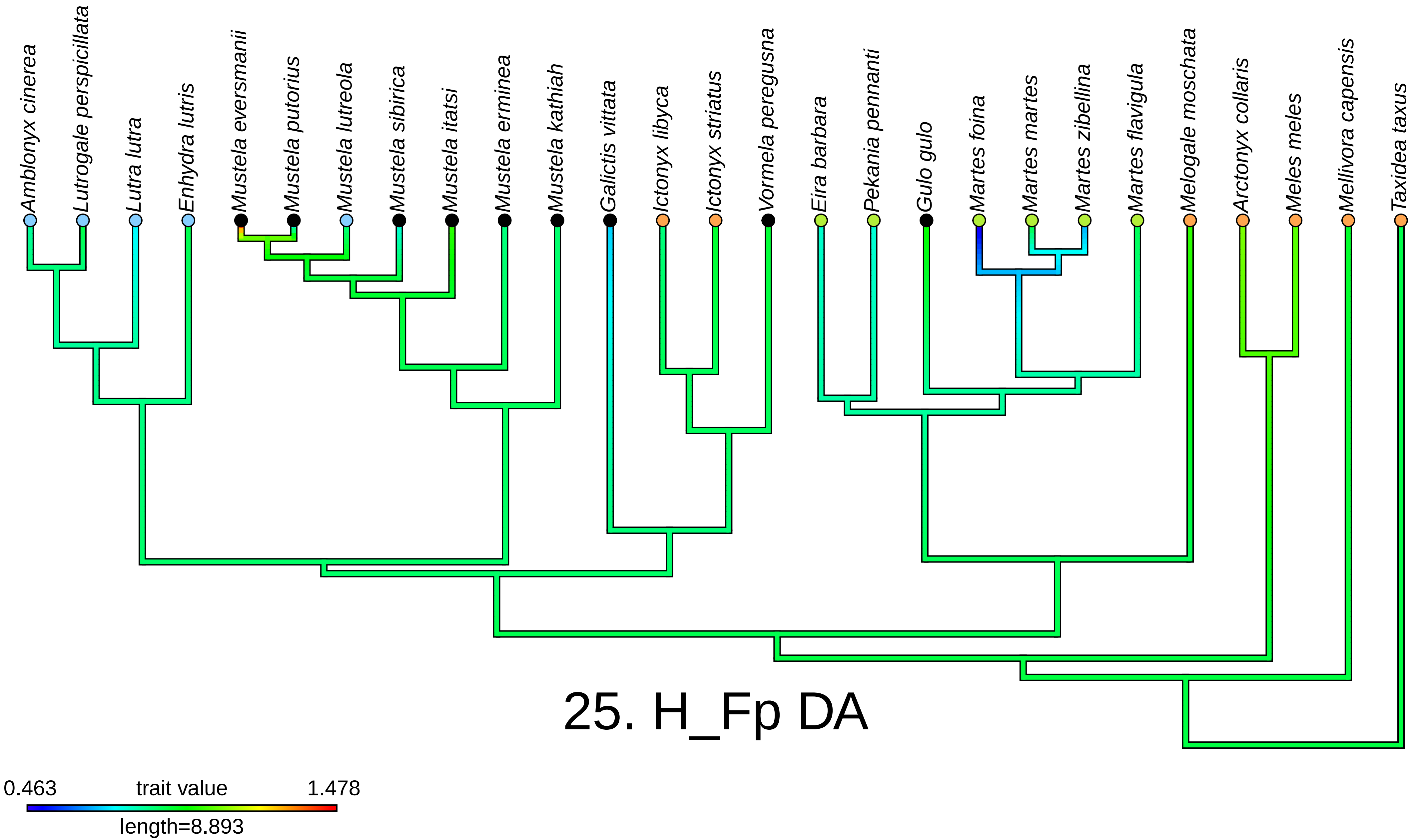
Amblonyx cinerea
Lutrogale perspicillata
Lutra lutra
Enhydra lutris
Mustela eversmanii
Mustela putorius
Mustela lutreola
Mustela sibirica
Mustela itatsi
Mustela erminea
Mustela kathiah
Galictis vittata
Ictonyx libyca
Ictonyx striatus
Vormela peregusna
Eira barbara
Pekania pennanti
Gulo gulo
Martes foina
Martes martes
Martes zibellina
Martes flavigula
Melogale moschata
Arctonyx collaris
Meles meles
Mellivora capensis
Taxidea taxus
25. H_Fp DA
0.463
trait value
1.478
length=8.893

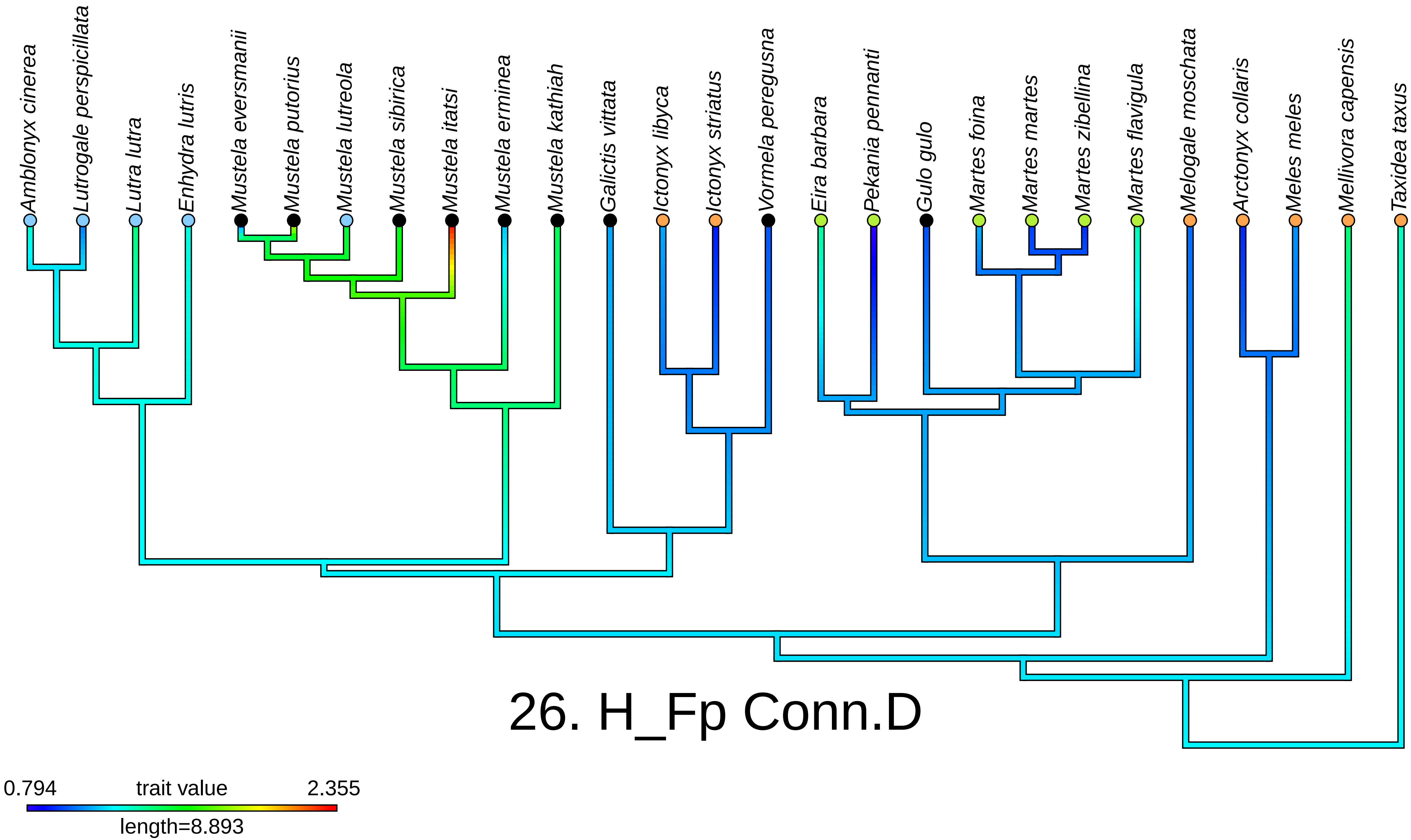
Amblonyx cinerea
Lutrogale perspicillata
Lutra lutra
Enhydra lutris
Mustela eversmanii
Mustela putorius
Mustela lutreola
Mustela sibirica
Mustela itatsi
Mustela erminea
Mustela kathiah
Galictis vittata
Ictonyx libyca
Ictonyx striatus
Vormela peregusna
Eira barbara
Pekania pennanti
Gulo gulo
Martes foina
Martes martes
Martes zibellina
Martes flavigula
Melogale moschata
Arctonyx collaris
Meles meles
Mellivora capensis
Taxidea taxus
0.794
trait value
2.355
length=8.893

26. H_Fp Conn.D

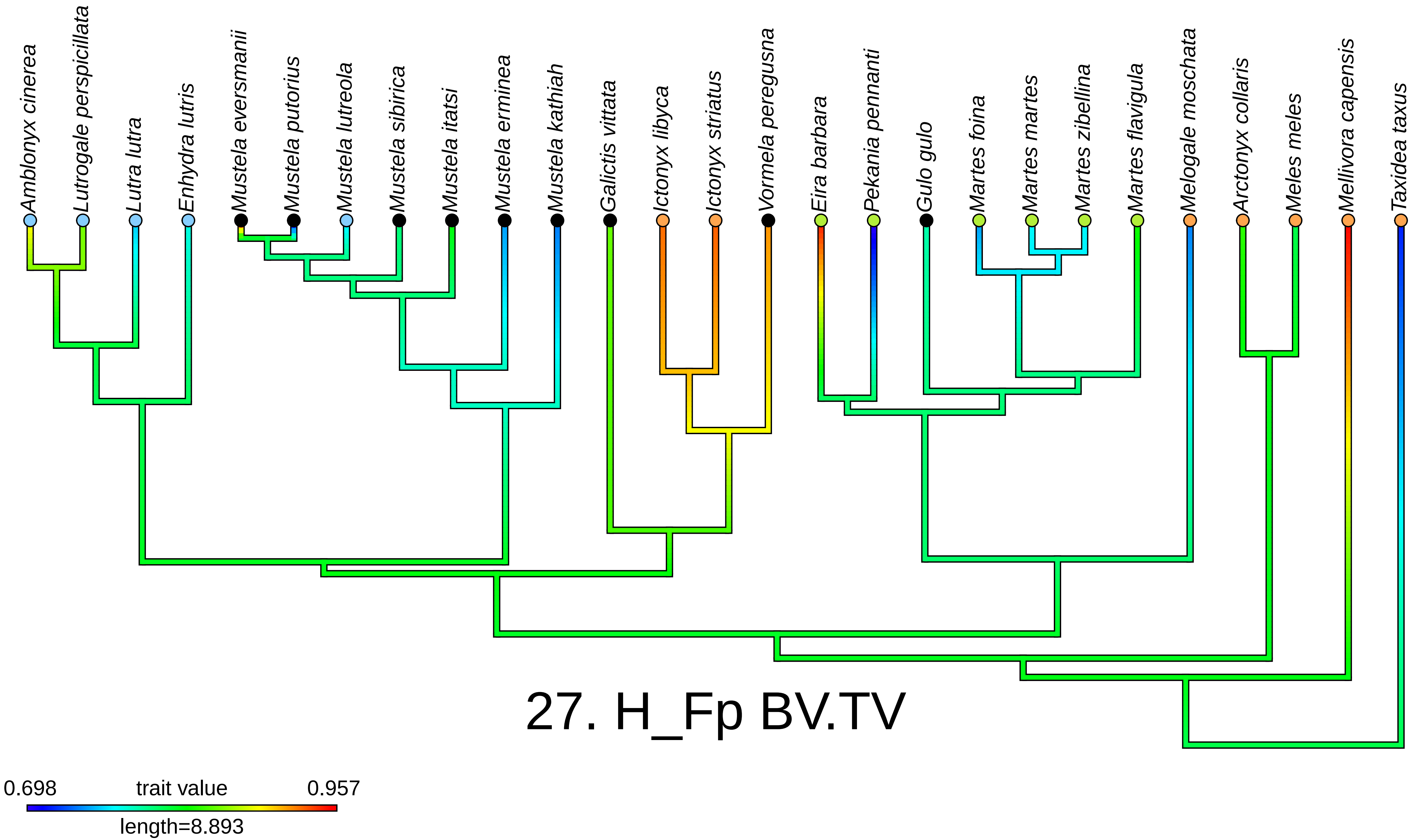

Amblonyx cinerea
Lutrogale perspicillata
Lutra lutra
Enhydra lutris
Mustela eversmanii
Mustela putorius
Mustela lutreola
Mustela sibirica
Mustela itatsi
Mustela erminea
Mustela kathiah
Galictis vittata
Ictonyx libyca
Ictonyx striatus
Vormela peregusna
Eira barbara
Pekania pennanti
Gulo gulo
Martes foina
Martes martes
Martes zibellina
Martes flavigula
Melogale moschata
Arctonyx collaris
Meles meles
Mellivora capensis
Taxidea taxus
27. H_Fp BV.TV
0.698
trait value
0.957
length=8.893

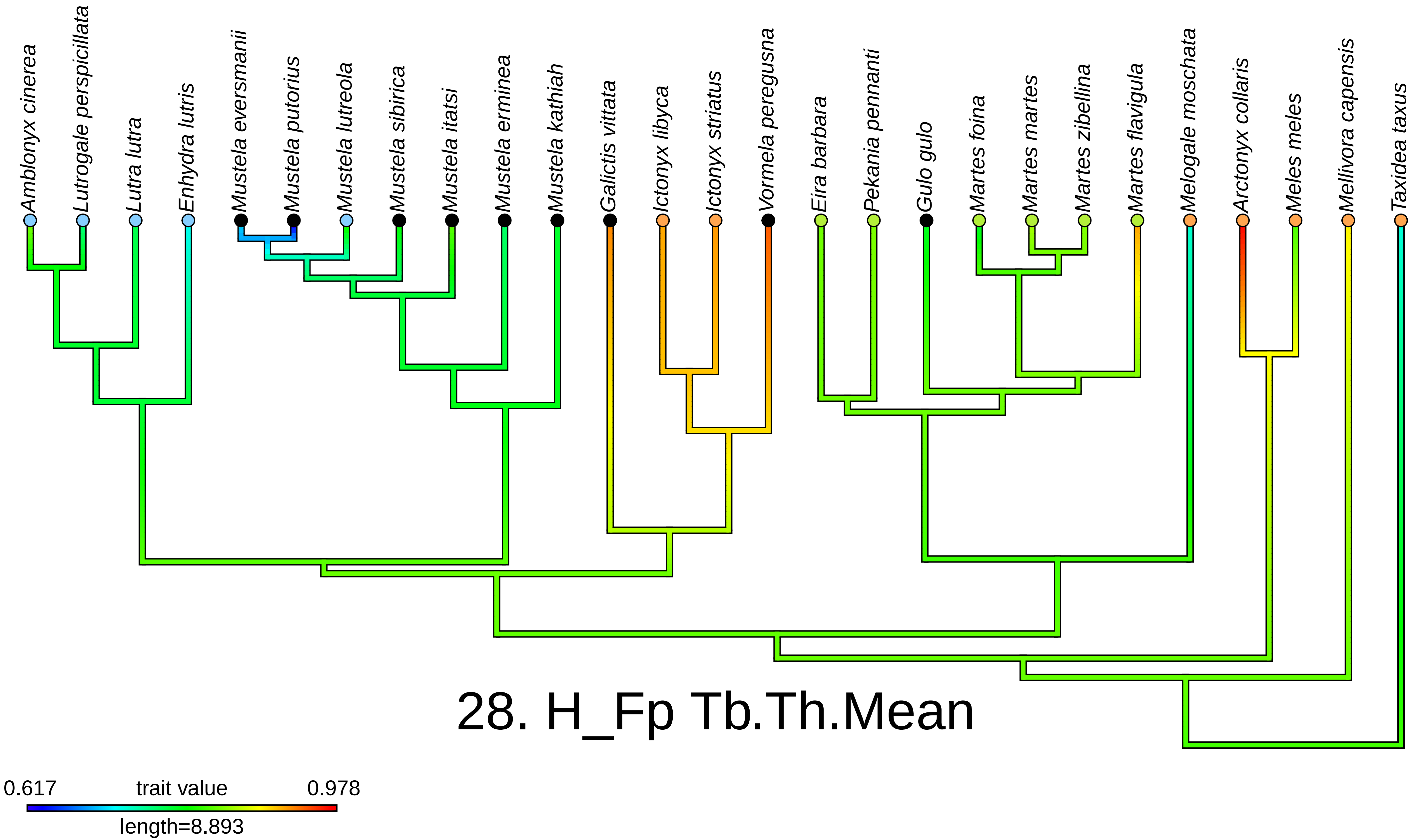

28. H_Fp Tb.Th.Mean

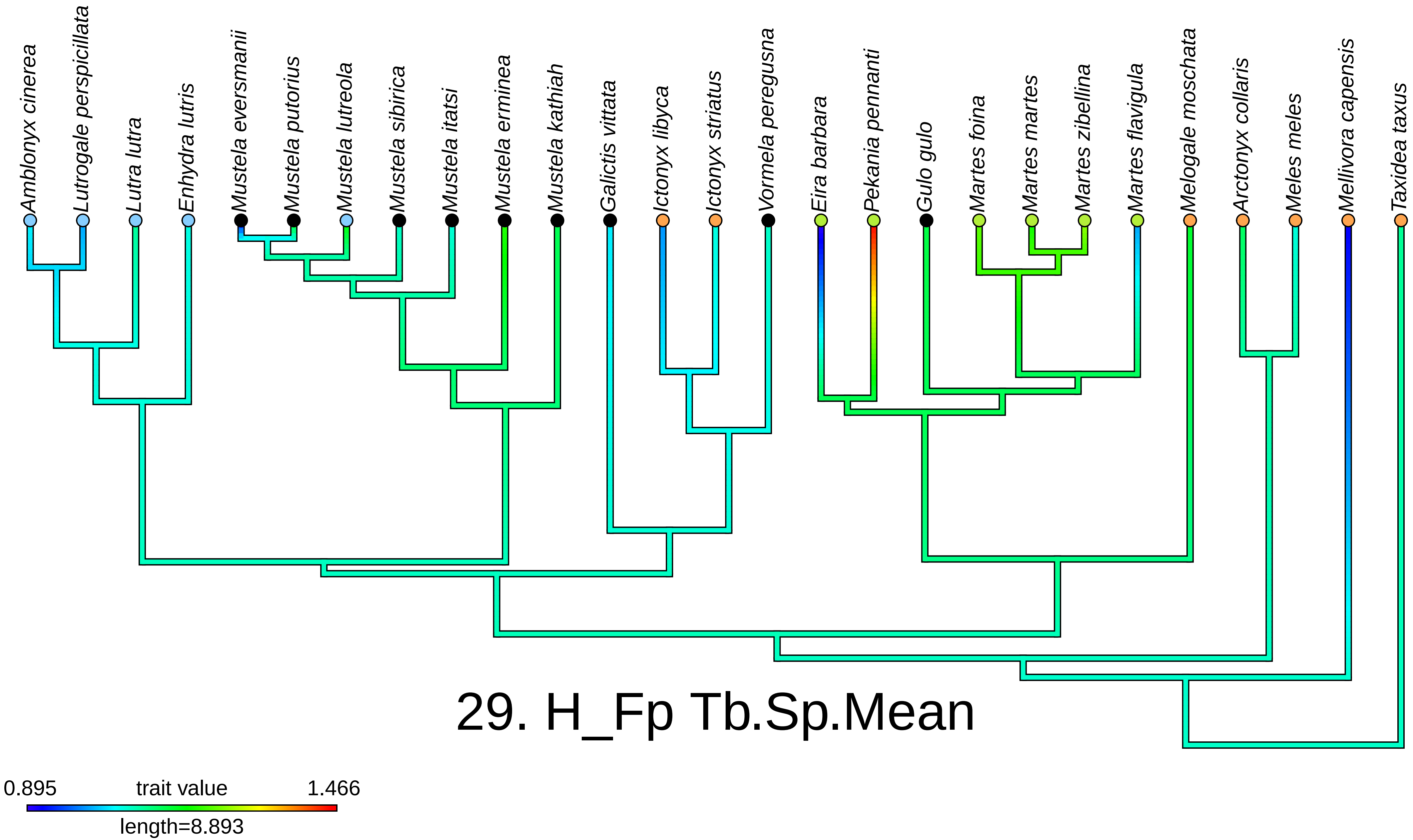

29. H_Fp Tb.Sp.Mean

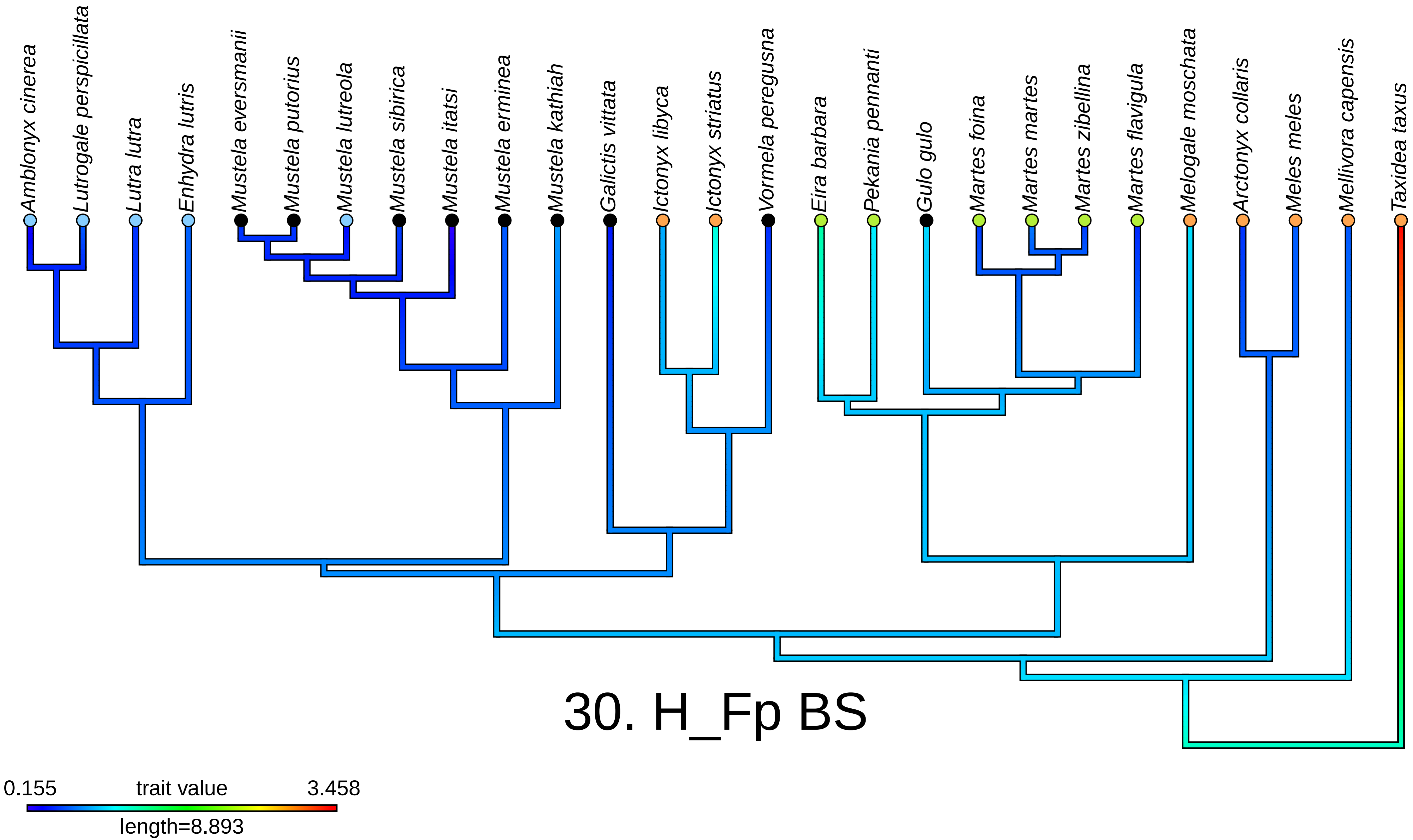
Amblonyx cinerea
Lutrogale perspicillata
Lutra lutra
Enhydra lutris
Mustela eversmanii
Mustela putorius
Mustela lutreola
Mustela sibirica
Mustela itatsi
Mustela erminea
Mustela kathiah
Galictis vittata
Ictonyx libyca
Ictonyx striatus
Vormela peregusna
Eira barbara
Pekania pennanti
Gulo gulo
Martes foina
Martes martes
Martes zibellina
Martes flavigula
Melogale moschata
Arctonyx collaris
Meles meles
Mellivora capensis
Taxidea taxus
0.155
trait value
3.458
length=8.893

30. H_Fp BS

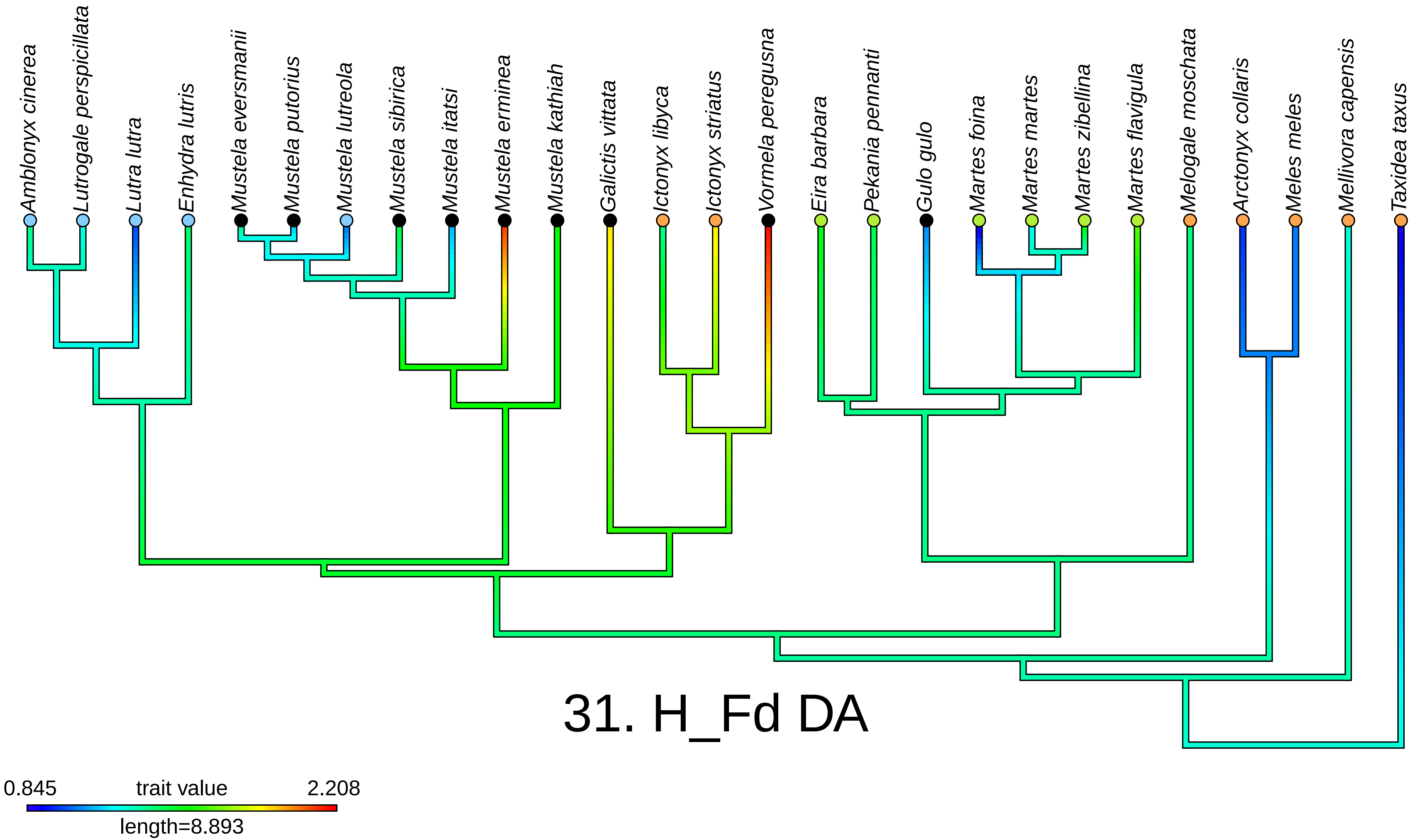
Amblonyx cinerea
Lutrogale perspicillata
Lutra lutra
Enhydra lutris
Mustela eversmanii
Mustela putorius
Mustela lutreola
Mustela sibirica
Mustela itatsi
Mustela erminea
Mustela kathiah
Galictis vittata
Ictonyx libyca
Ictonyx striatus
Vormela peregusna
Eira barbara
Pekania pennanti
Gulo gulo
Martes foina
Martes martes
Martes zibellina
Martes flavigula
Melogale moschata
Arctonyx collaris
Meles meles
Mellivora capensis
Taxidea taxus
0.845
trait value
2.208
length=8.893

31. H_Fd DA

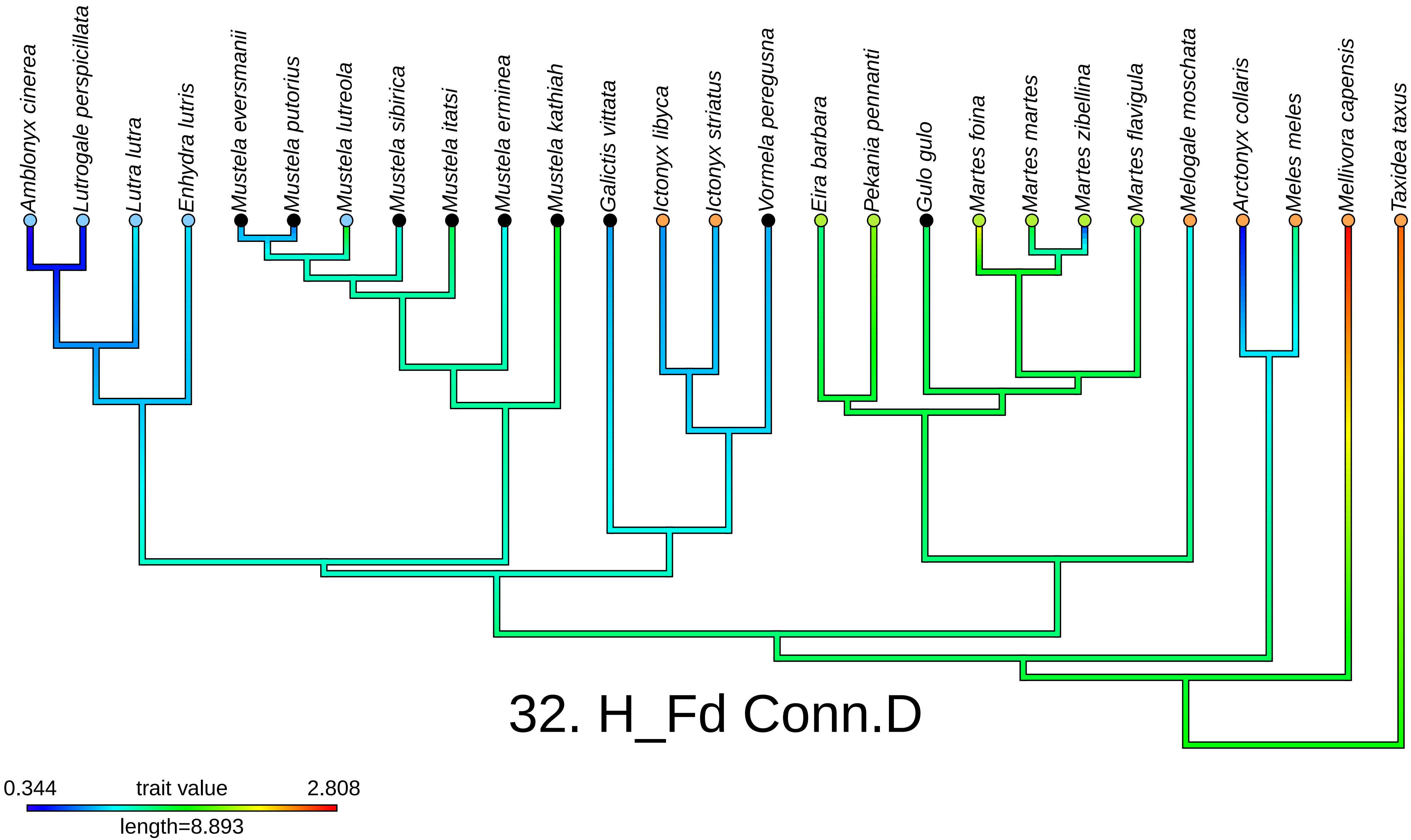

Amblonyx cinerea
Lutrogale perspicillata
Lutra lutra
Enhydra lutris
Mustela eversmanii
Mustela putorius
Mustela lutreola
Mustela sibirica
Mustela itatsi
Mustela erminea
Mustela kathiah
Galictis vittata
Ictonyx libyca
Ictonyx striatus
Vormela peregusna
Eira barbara
Pekania pennanti
Gulo gulo
Martes foina
Martes martes
Martes zibellina
Martes flavigula
Melogale moschata
Arctonyx collaris
Meles meles
Mellivora capensis
Taxidea taxus
0.344
trait value
2.808
length=8.893

32. H_Fd Conn.D

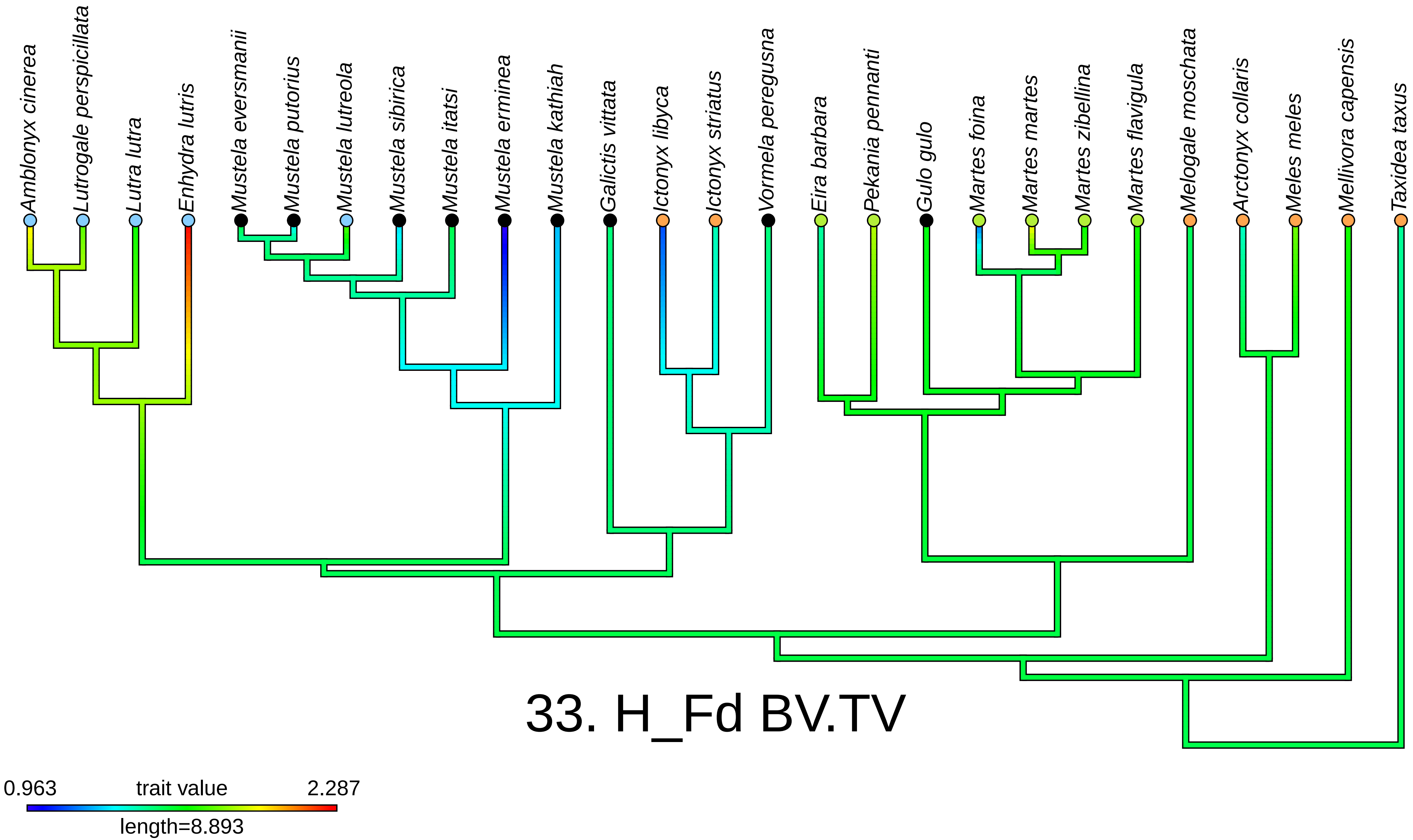
Amblonyx cinerea
Lutrogale perspicillata
Lutra lutra
Enhydra lutris
Mustela eversmanii
Mustela putorius
Mustela lutreola
Mustela sibirica
Mustela itatsi
Mustela erminea
Mustela kathiah
Galictis vittata
Ictonyx libyca
Ictonyx striatus
Vormela peregusna
Eira barbara
Pekania pennanti
Gulo gulo
Martes foina
Martes martes
Martes zibellina
Martes flavigula
Melogale moschata
Arctonyx collaris
Meles meles
Mellivora capensis
Taxidea taxus
33. H_Fd BV.TV
0.963
trait value
2.287
length=8.893

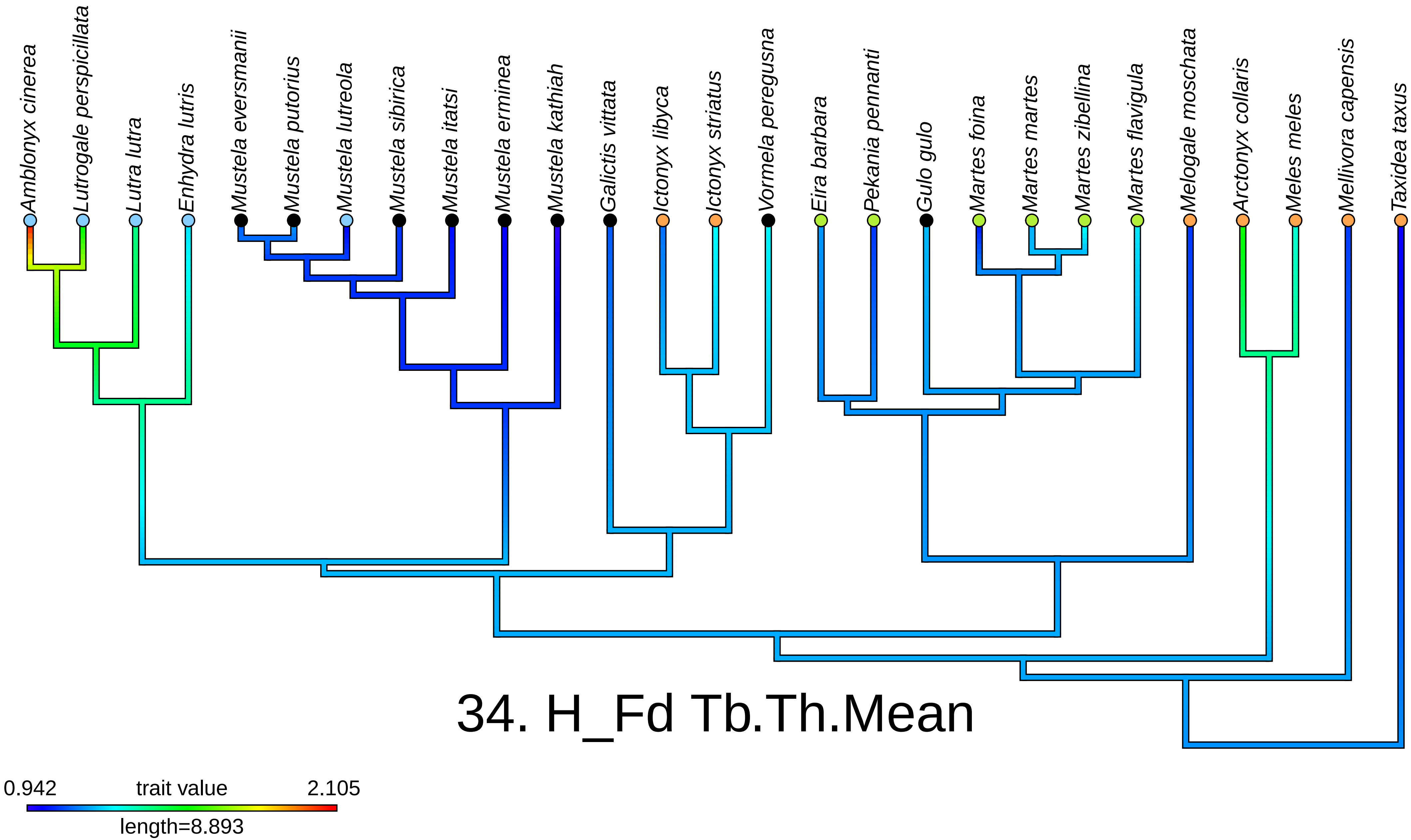

34. H_Fd Tb.Th.Mean

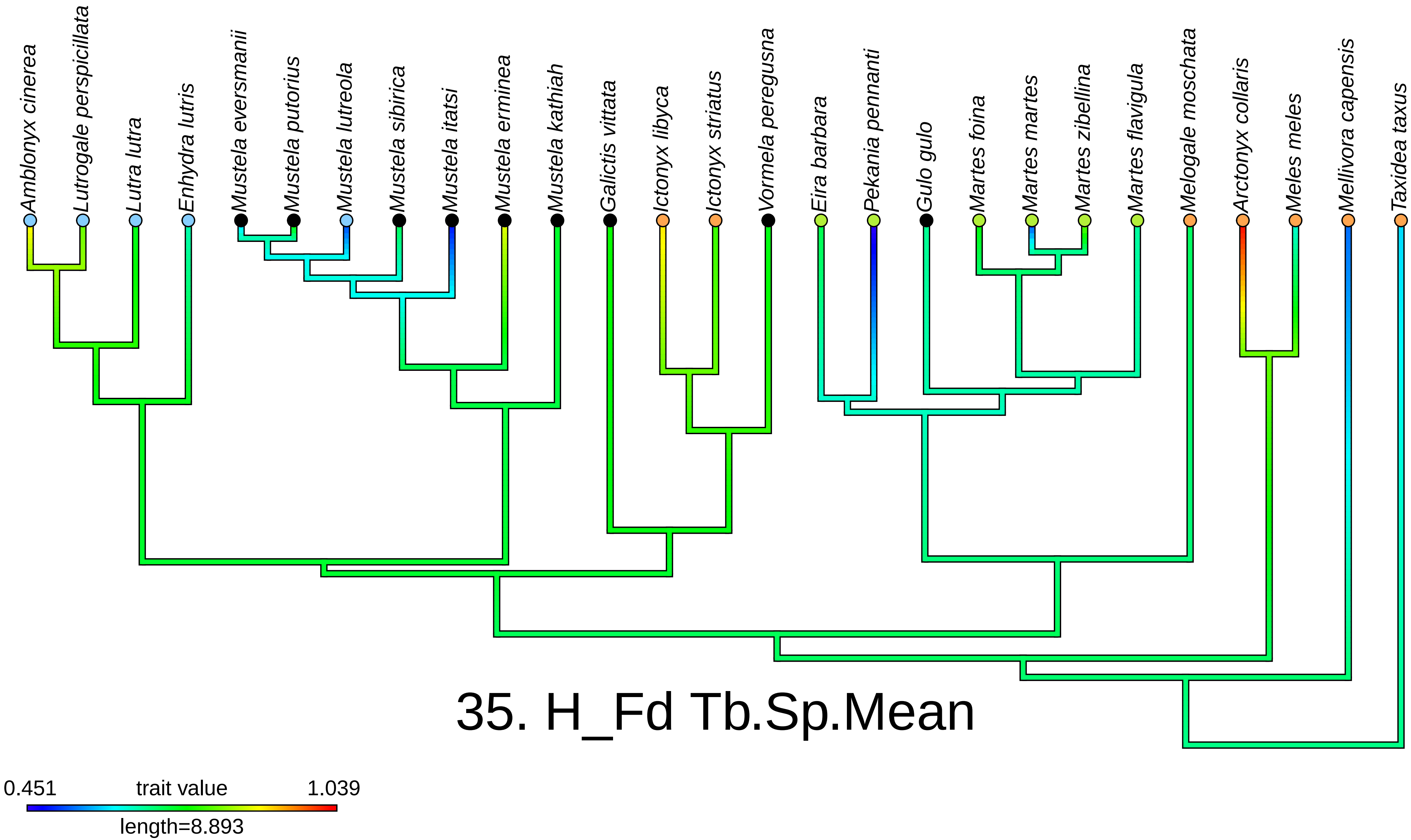

35. H_Fd Tb.Sp.Mean

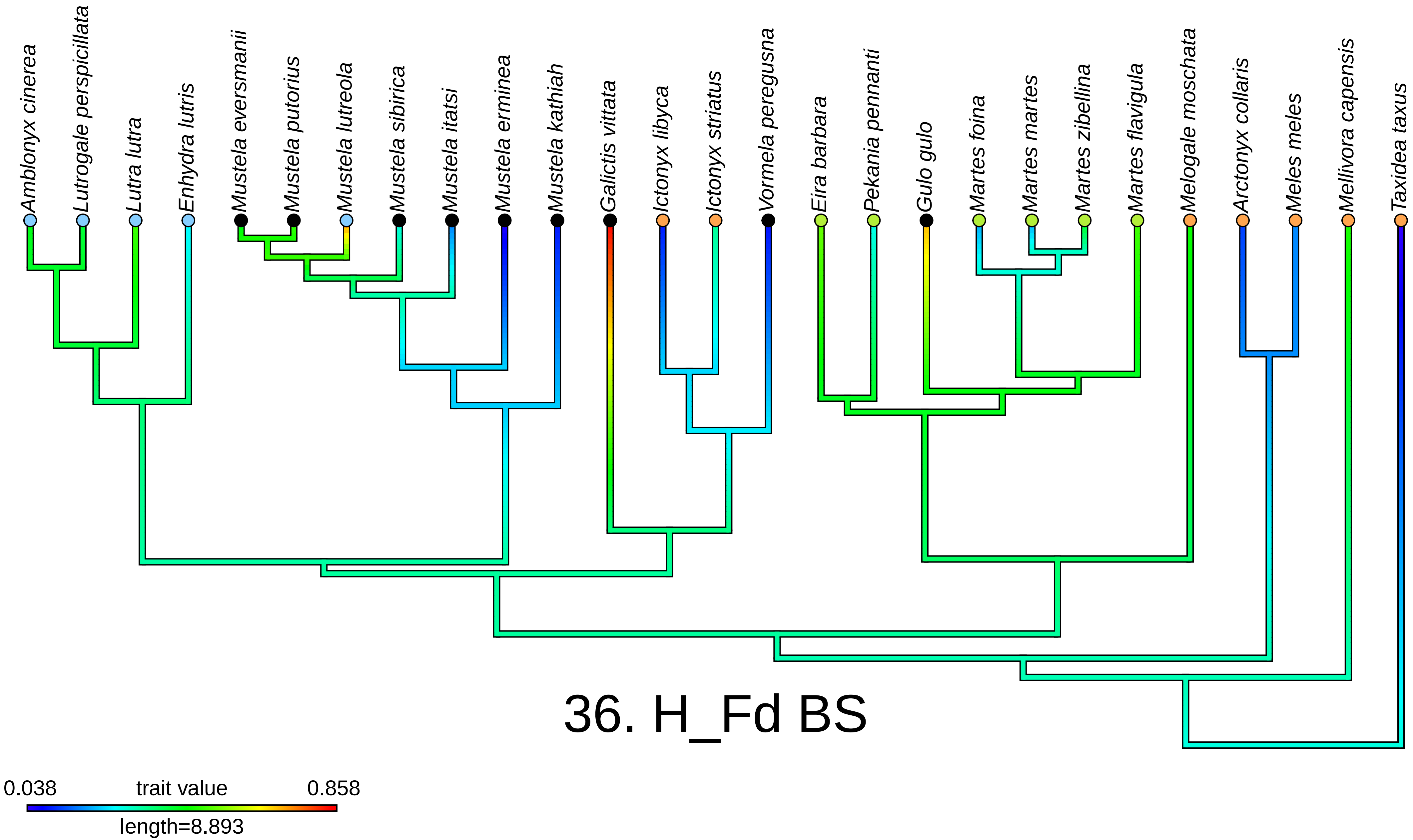
Amblonyx cinerea
Lutrogale perspicillata
Lutra lutra
Enhydra lutris
Mustela eversmanii
Mustela putorius
Mustela lutreola
Mustela sibirica
Mustela itatsi
Mustela erminea
Mustela kathiah
Galictis vittata
Ictonyx libyca
Ictonyx striatus
Vormela peregusna
Eira barbara
Pekania pennanti
Gulo gulo
Martes foina
Martes martes
Martes zibellina
Martes flavigula
Melogale moschata
Arctonyx collaris
Meles meles
Mellivora capensis
Taxidea taxus
36. H_Fd BS
0.038
trait value
0.858
length=8.893
