# Supplementary material for: Trabecular bone architecture in the stylopod epiphyses of mustelids (Mammalia, Carnivora)
Source: R Soc Open Sci. 2019 Oct 23;6(10):190938. doi: 10.1098/rsos.190938 (PMC6837213; doi:10.1098/rsos.190938)
Supplement: SM 8 [file rsos190938supp8.pdf]

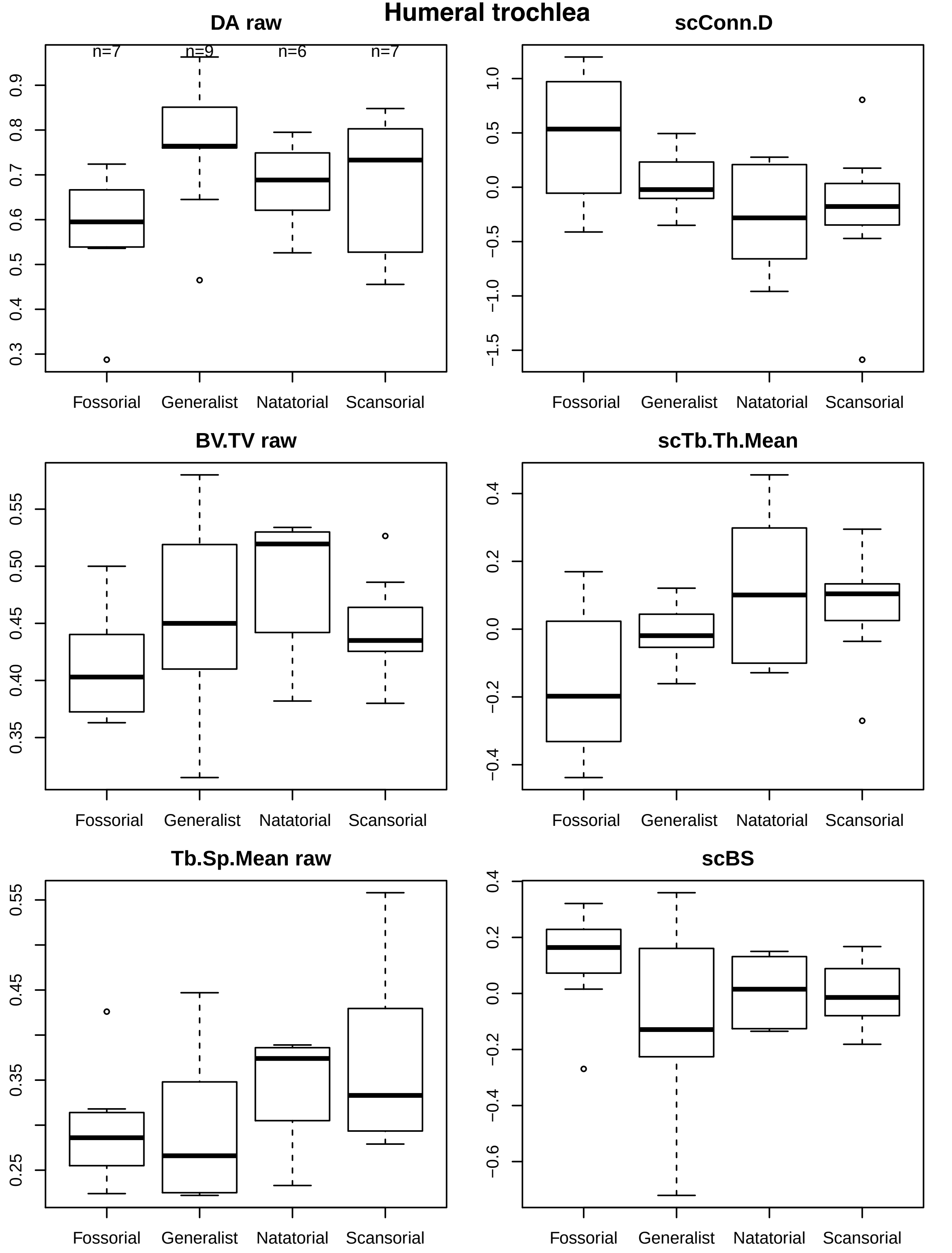

Humeral trochlea
DA raw
n=7
n=9
n=6
n=7
scConn.D
BV.TV raw
scTb.Th.Mean
Tb.Sp.Mean raw
scBS
Fossorial
Generalist
Natatorial
Scansorial

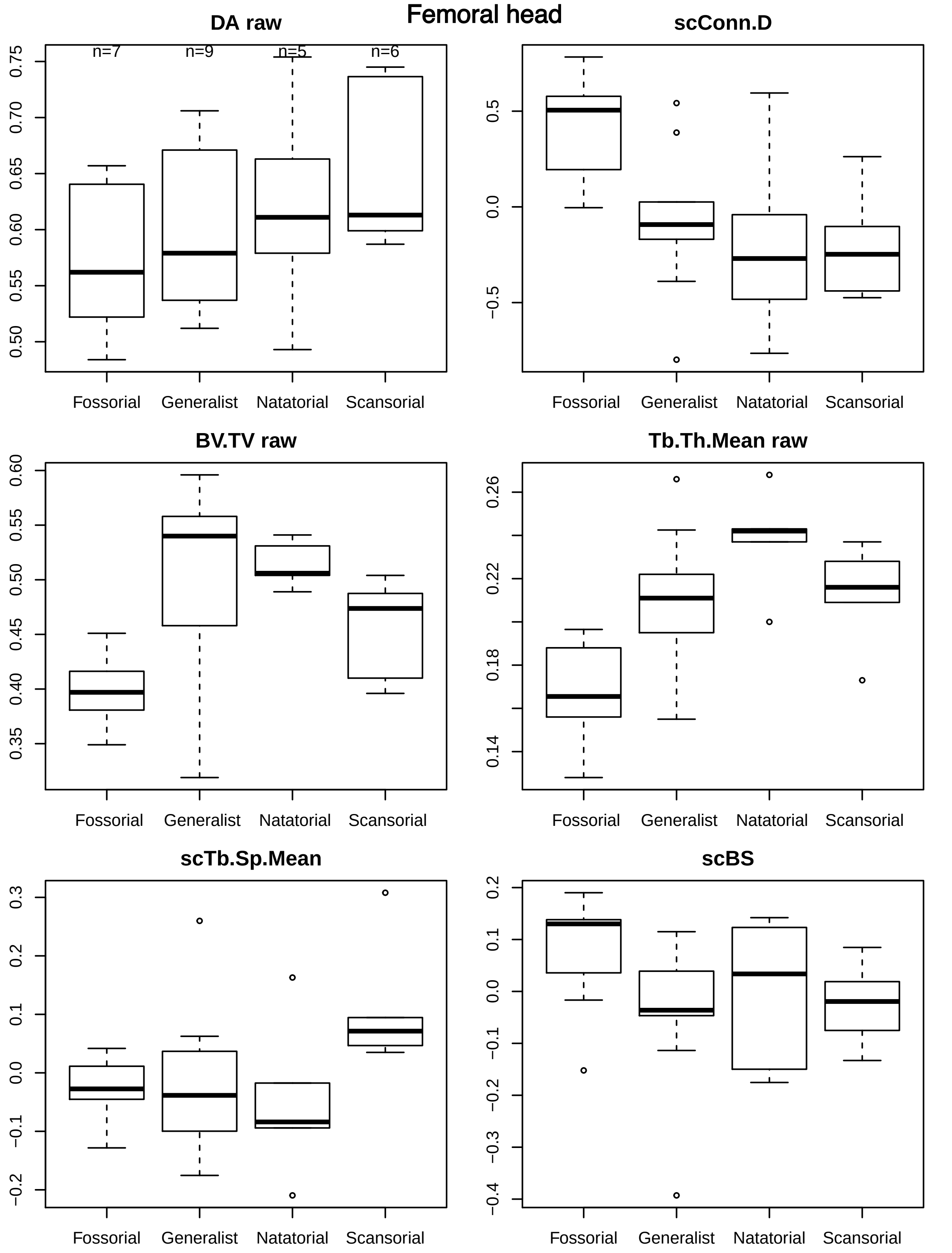
Femoral head
DA raw
n=7
n=9
n=5
n=6
0.50
0.55
0.60
0.65
0.70
0.75
scConn.D
-0.5
0.0
0.5
BV.TV raw
0.35
0.40
0.45
0.50
0.55
0.60
Tb.Th.Mean raw
0.14
0.18
0.22
0.26
scTb.Sp.Mean
-0.2
-0.1
0.0
0.1
0.2
0.3
scBS
-0.4
-0.3
-0.2
-0.1
0.0
0.1
0.2
Fossorial
Generalist
Natatorial
Scansorial

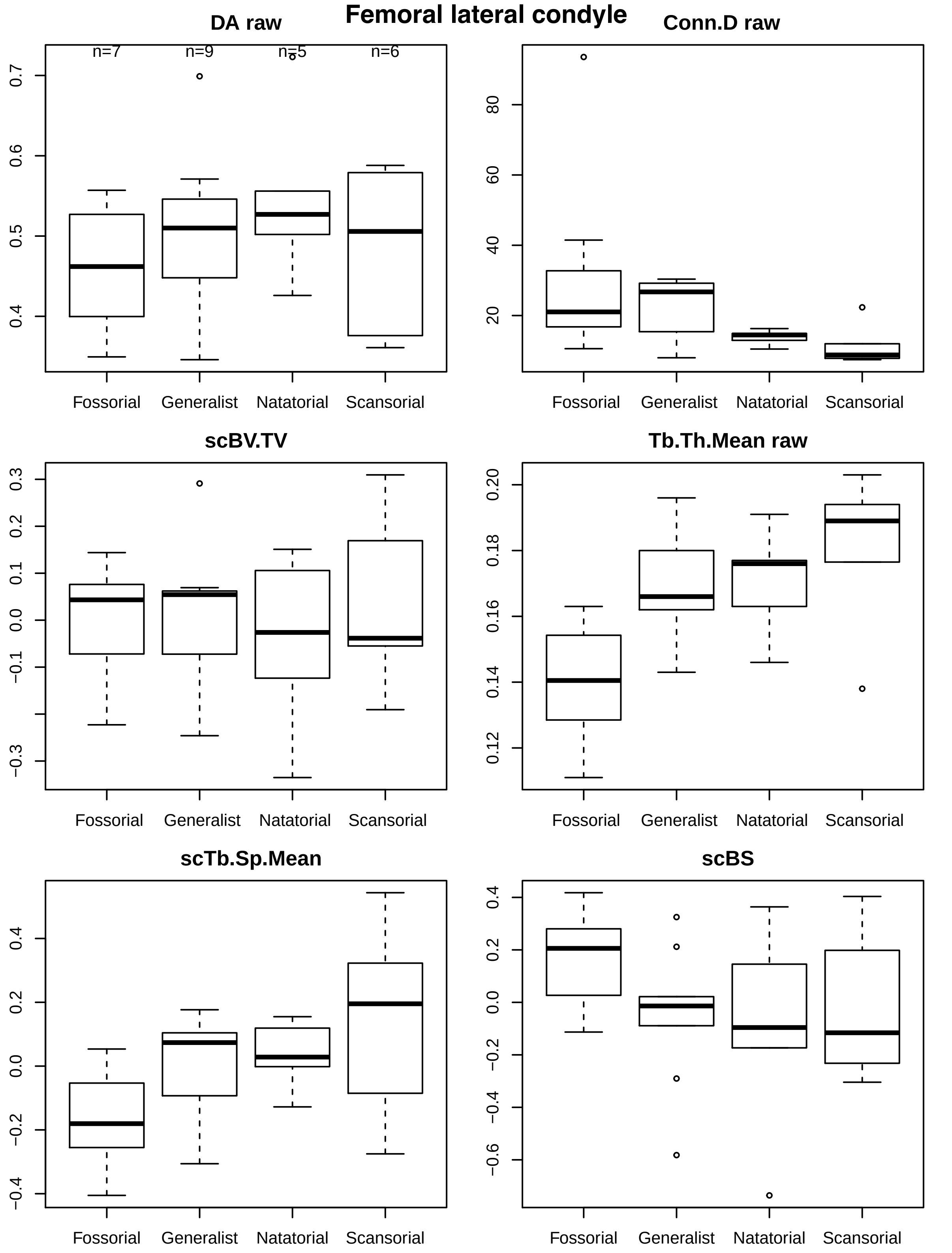

Femoral lateral condyle
DA raw
n=7
n=9
n=5
n=6
Conn.D raw
scBV.TV
Tb.Th.Mean raw
scTb.Sp.Mean
scBS
Fossorial
Generalist
Natatorial
Scansorial
