# Supplementary material for: Trabecular bone architecture in the stylopod epiphyses of mustelids (Mammalia, Carnivora)
Source: R Soc Open Sci. 2019 Oct 23;6(10):190938. doi: 10.1098/rsos.190938 (PMC6837213; doi:10.1098/rsos.190938)
Supplement: SM 11 [file rsos190938supp11.docx]

Supplementary Online Material for:

Trabecular bone architecture in the stylopod epiphyses of mustelids (Mammalia, Carnivora)

Amson, E.^1^ and Kilbourne, B.M.^1^

^1^Museum für Naturkunde, Leibniz-Institut für Evolutions- und Biodiversitätsforschung, Berlin, Germany

SOM 11. pPCA plot of the loadings (see also Fig. 5 and SOM 10).
